# Targeting efferocytosis for tissue regeneration: From microenvironment reprogramming to clinical translation

**DOI:** 10.7150/thno.126081

**Published:** 2026-01-08

**Authors:** Yunzhu Li, Peiyu Li, Jiayi Song, Xue Zhang, Haitao Xiao, Ru Wang, Zhenyu Duan, Kui Luo, Xuewen Xu

**Affiliations:** 1 Department of Plastic and Burn Surgery, Department of Radiology, Huaxi MR Research Center (HMRRC), Institution of Radiology and Medical Imaging, Rehabilitation Therapy, Institute of Breast Health Medicine, Frontiers Science Center for Disease-Related Molecular Network, State Key Laboratory of Biotherapy, West China Hospital, Sichuan University, Chengdu 610041, China.; 2 Psychoradiology Key Laboratory of Sichuan Province, Key Laboratory of Transplant Engineering and Immunology, NHC, and Research Unit of Psychoradiology, Chinese Academy of Medical Sciences, Chengdu 610041, China.

**Keywords:** efferocytosis, tissue regeneration, macrophage, inflammation resolution, biomaterials

## Abstract

Efferocytosis, phagocytic clearance of apoptotic cells (ACs), is an essential biological process that resolves inflammation and regulates tissue regeneration in various organ systems. Through removal of apoptotic cell debris, efferocytosis attenuates secondary necrosis and dampens the release of damage-associated molecular patterns (DAMPs). More importantly, it can reprogram phagocytes towards a pro-reparative phenotype via the secretion of anti-inflammatory mediators, metabolic rewiring, and the production of growth factors. There are four closely regulated stages in the entire process: “find-me” signal-mediated phagocyte recruitment, recognition of ACs via “eat-me” signals, AC internalization via Rho GTPase-dependent actin remodeling, and phagolysosomal degradation of ACs by either canonical or light chain 3 (LC3)-associated phagocytosis (LAP). In a repair context, efferocytosis may refer to the clearance of dying cells during various tissue repair processes, such as wound healing, liver injury, myocardial infarction, intestinal damage, kidney injury and muscle injury. Efferocytosis regulates inflammation resolution, stem/progenitor cell activation, extracellular matrix remodeling, and angiogenesis to coordinate tissue repair. Chronic pathology (e.g., diabetic ulcers, fibrosis) induced by dysfunctional efferocytosis results from accumulation of non-phagocytosed ACs that maintain inflammation and impair regeneration. Therapeutic strategies targeting dysfunctional efferocytosis have been developed, encompassing active pharmaceutical ingredients, biologics, and biomaterials-assisted therapeutic modalities. Despite promising outcomes from preclinical studies, challenges still exist in the spatiotemporal control and clinical translation of these therapeutic strategies. Future research could focus on the multi-omics integration and smart biomaterial development to dynamically modulate efferocytosis during different disease phases.

## 1. Introduction

Tissue regeneration is a well-conserved process in evolution. When activated, the function/structure of damaged tissues are restored by switching on a series of cellular processes for regeneration, which involve damage sensing, immune modulation, progenitor cell mobilization, extracellular matrix (ECM) deposition and remodeling. This regeneration program is dampened in patients with chronic inflammatory and autoimmune diseases. Specifically, in terms of prevalent comorbidities, patients with type 2 diabetes, hypertension and coronary artery disease often exhibit pathological fibrosis or regenerative failure.

Efferocytosis or phagocytic clearance of apoptotic cells (ACs) is an essential process for maintaining tissue homeostasis by phagocytes 1,2. Phagocytes can be classified into three groups: professional phagocytes (e.g., macrophages, neutrophils, dendritic cells (DCs)) with high phagocytic ability, nonprofessional phagocytes (e.g., epithelial cells, fibroblasts) with incompetent engulfment ability, and specialized phagocytes (e.g., Sertoli cells in testes) with both tissue-specific functions and phagocytotic ability 3. Evidence has suggested that efferocytosis confers dual regenerative benefits after injury or disease progression: (1) preventing secondary necrosis and inflammatory damage from leaked cellular contents from ACs, and (2) reprogramming phagocytes into a pro-resolution phenotype through the secretion of anti-inflammatory mediators and the regulation of repair-promoting transcriptional pathways 4,5. Unfortunately, impaired efferocytosis is often observed under chronic non-healing conditions, which results in the accumulation of secondary necrotic cells. The accumulation of necrotic cells perpetuates inflammatory cascades and simultaneously weakens pro-regenerative signals derived from ACs clearance in the tissue, creates a hostile microenvironment for tissue reconstruction 6-8.

The impact of the microenvironment created from efferocytosis on regenerative niches is rarely systematically elaborated and the effect of efferocytosis on key effector cells (e.g., stem cells, fibroblasts) is insufficiently elucidated in previous review articles 9. We systematically examine mechanistic insights for efferocytosis in coordinating inflammation resolution with tissue regeneration through spatiotemporal metabolic reprogramming and intercellular crosstalk in this review article. We reveal mechanisms of efferocytosis in organ/tissue repair and highlight its dual role in chronic diseases including promoting tissue repair and driving pathological fibrosis under dysregulated conditions. After summarizing current efferocytosis-targeting strategies, we critically analyze barriers for targeting efferocytosis and provide our insights into future directions in addressing these barriers.

## 2. Efferocytosis processes and their regulation

**Figure 1:**
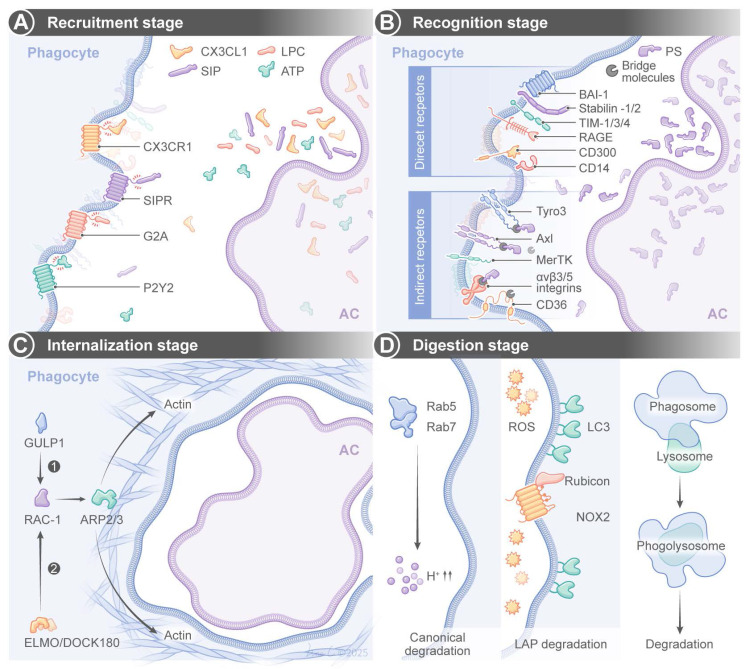
** Efferocytosis processes and their regulation.** Efferocytosis is achieved through four sequential stages: (A) recruitment, (B) recognition, (C) internalization and (D) digestion. SIP sphingosine-1-phosphate, SIPR: sphingosine-1-phosphate receptor, LPC: lysophosphatidylcholine, ATP: adenosine triphosphate, PS: phosphatidylserine, BAI-1: brain angiogenesis inhibitor-1, TIM: T-cell immunoglobulin mucin, RAGE: receptorfor advanced glycation end products, GULP1: engulfment adaptor protein 1, ARP2/3 actin-related protein 2/3, ELMO/DOCK180: the complex of the engulfment and cell motility protein and the dedicator of cytokinesisprotein, ROS: reactive oxygen species, LC3: autophagy-related protein 1A/1B-light chain 3, NOX2: nicotinamide adenine dinucleotide phosphate oxidase-2.

Efferocytosis is a tightly regulated multistep process, and it has a few key stages (**Figure 1**). The first stage is the “recruitment phase”, in which ACs release “find-me” signals to attract phagocytes via receptors such as CX3 C-chemokine receptor 1 (CX3CR1), G2 accumulation protein receptor (G2A), or P2Y purinoceptor 2 (P2Y2). The signals include chemokines (e.g., CX3 C-chemokine ligand 1(CX3CL1)/CX C-chemokine ligand 12 (CXCL12)), lipids (e.g., sphingosine 1-phosphate (S1P)/lysophosphatidylcholine (LPC)), or nucleotides (e.g., adenosine triphosphate (ATP)) 10-12. The second stage is the “recognition phase”, in which “eat-me” signals from ACs (e.g., phosphatidylserine (PS), calreticulin) bind directly to phagocyte receptors (e.g., T-cell immunoglobulin and mucin-domain (TIM) family, adhesion G protein-coupled receptor B1 (aDGrB1; also known as BAI1)) or indirectly to receptors like the Tyro3-Axl-MerKT (TAM) family or integrins via bridging molecules (e.g., growth arrest-specific gene 6 (Gas6), milk fat globule-epidermal growth factor 8 (MFG-E8)) 13-25. Complementary to these pro-phagocytic signals, healthy cells actively maintain “don't-eat-me” signals to prevent inappropriate engulfment by phagocytes. CD47 is the most well-characterized don't-eat-me signal and it is a transmembrane glycoprotein that interacts with signal regulatory protein α (SIRPα) in phagocytes to deliver inhibitory signals through tyrosine phosphatase SHP-1/2, thereby suppressing phagocytosis 26. CD24 is another “don't-eat-me” signal, and it binds to Siglec-10 on phagocytes to inhibit engulfment of aging neutrophils and cancer cells 27. The dynamic balance between “eat-me” and “don't-eat-me” signals critically determine the efferocytosis efficiency under both normal physiology and diseased conditions.

The third stage is the internalization phase, in which receptor engagement activates Ras homologous guanosine-5'-triphosphatases (Rho GTPases) (e.g., Rac family small GTPase 1 (s)) via adaptors (e.g., engulfment and cell motility protein (ELMO), dedicator of cytokinesis 1 (DOCK180), engulfment adaptor PTB domain containing 1 (GULP1)) to trigger actin polymerization via actin-related protein 2/3 complex (ARP2/3) and WASP-family verprolin-homologous protein 1 (WAVE1), leading to the formation of a phagocytotic cup to engulf ACs 19,28-31. Finally, in the digestion phase, phagosomes fuse with lysosomes to form phagolysosomes for enzymatic degradation of ACs via two pathways. The canonical degradation pathway involves phagosome maturation. Phagosomes mature via an increasingly acidic membrane-bound structure, such as Ras-related in brain protein 5 (Rab5) and Rab7 32,33. The other pathway is LC3-associated phagocytosis (LAP). Class III phosphatidylinositol 3-kinase (PI3KC3), Rubicon, and nicotinamide adenine dinucleotide phosphate oxidase 2 (NOX2)-generated reactive oxygen species (ROS) are actively engaged in LC3-lipidation 34-37.

## 3. Roles of efferocytosis in tissue repair

In addition to resolving cellular debris, efferocytosis facilitates tissue repair and regeneration by modulating the immune microenvironment, activating pro-regenerative programs, and coordinating remodeling of the ECM to restore tissue homeostasis through four closely connected steps: injury sensing, inflammation resolution, pro-regeneration cell activation, and ECM formation/remodeling 38. 1) Injury sensing. Efferocytosis is primed by sensing apoptotic “find-me” signals to recruit macrophages toward injury sites. On the other hand, apoptotic cells also release PS as an essential “eat-me” signal on their surface 10,11. This balancing signaling network enables apoptotic cells to be specifically recognized while protecting surrounding normal tissues from unnecessary immune stimulation. 2) Inflammation resolution. Efferocytosis attenuates pro-inflammatory environment by modulating macrophage metabolism. When engulfing apoptotic cells, macrophages switch to a metabolically oxidative state to drive the formation of multiple macrophage subpopulations with modulated pro-inflammatory (interleukin-1β (IL-1β), tumor necrosis factor-α (TNF-α)) and anti-inflammatory (mannose receptor C type 1 (CD206, or MRC1), transforming growth factor-β (TGF-β) and Arginase 1 (Arg1)) markers 39,40. 3) Pro-repair cell activation. Efferocytosis converts phagocytes into regenerative effector cells. Metabolites released by apoptotic cells (e.g., arginine, nucleotides) activate mechanistic targets of rapamycin complex 2 (mTORC2) signaling to promote macrophage proliferation and functional adaptation 41,42. Moreover, efferocytotic macrophages also produce and release growth factors such as vascular endothelial growth factor (VEGF) and platelet-derived growth factor (PDGF), which stimulate angiogenesis and fibroblasts to mediate connective tissue repair 43,44. In addition to these functions, metabolic reprogramming in macrophages provides energy supply for stem cell niches and promotes stem cell proliferation 45. Furthermore, efferocytosis directly activates stem cell differentiation through secreted factors, such as TGF-β and Wingless/INT-1 (Wnt) ligands, which prime lineage-specific programs 46. Consistently, immunomodulatory molecules produced by stem cells (e.g., prostaglandin E2 (PGE2), tumor necrosis factor-stimulated gene 6 (TSG-6)) increase the ability of phagocytes to engulf apoptotic cells, thus amplifying the regenerative program 47. 4) ECM formation and remodeling. Efferocytosis counteracts the dynamics of the ECM through a dual-role regulatory mechanism that promotes both matrix synthesis and matrix degradation. Efferocytotic macrophages release interleukin-1α (IL-1α) and osteopontin, which mediate fibroblasts activation for depositing and organizing fibrillar collagen into a provisional repair matrix 48. Conversely, efferocytosis can also regulate collagen degradation and structural remodeling by reparative macrophages which are associated with source-level suppression of pro-fibrotic signaling after ACs resolution (**Table 1**) 49.

### 3.1 Efferocytosis in wound healing

Normal wound healing is a well-coordinated process consisting of four well-defined stages: hemostasis, inflammation, proliferation, and remodeling 50,51. When an injury occurs, neutrophils, the first line of recruited circulating leukocytes, arrive at the wound site and establish defenses against pathogens through the release of proteases and ROS 52,53. Neutrophils are indispensable for killing invading pathogens; however, their massive influx into the wound bed is followed by a tightly regulated death of neutrophils through a process of efferocytosis 54. Phagocytic scavenging of apoptotic neutrophils can prevent secondary necrosis and persistent inflammation during the resolution phase 54. This process is regulated by a sophisticated molecular interplay between apoptotic neutrophils and macrophages. The matricellular protein, cellular communication network factor 1 (CCN1), has been identified as a regulator of this process. CCN1 can establish a bridge between PS, an evolutionarily conserved “eat-me” signal exposed on apoptotic neutrophils, and integrins αvβ3/αvβ5 on macrophage surface. Interaction between neutrophils and macrophages induced RAC1-dependent cytoskeletal remodeling, promoted phagosome formation and induced debris internalization 55. Importantly, mechanical bridging is not the only function of CCN1, and it can regulate metabolic reprogramming of macrophages.

Macrophages display high levels of functional plasticity during wound healing, and their phenotype switches dynamically between pro-inflammatory M1 and anti-inflammatory M2 in a microenvironment-dependent manner 56. Efferocytosis markedly modulates this polarization. Sequestration of apoptotic neutrophils supply macrophages with long-chain fatty acids that can be used to drive nicotinamide adenine dinucleotide positive (NAD+)-dependent metabolic pathways, thereby promoting oxidative phosphorylation and suppressing glycolysis. This change in metabolism upregulates interleukin-10 (IL10) expression through signal transducer and activator of transcription 3 (STAT3) and potentiates M2 polarization while suppressing TNF-α expression through the nuclear factor kappa-light-chain-enhancer of activated B cells (NF-κB) 57. The glycoprotein MFG-E8 can further amplify this switch by acting as a molecular glue that bridges PS on apoptopic cells and αvβ3/αvβ5 integrins on macrophages. Transcriptomic analyses have shown upregulation of MFG-E8 during skin repair, and we have recently demonstrated that debris clearance is defective, TNF-α/IL10 ratio is increased and wound healing is delayed in mice deficient for MFG-E8 (MFG-E8-/-) 58-60. In addition to promoting phagocytosis, MFG-E8 directly triggers M2 polarization by activating phosphoinositide 3-kinase/protein kinase B (PI3K/AKT) signaling, thus promoting the production of basic fibroblast growth factor (bFGF) and enhancing fibroblast migration and collagen deposition 61. In addition, neutrophil-derived intercellular adhesion molecule 1 (ICAM-1) optimizes efferocytosis through the activation of the fibrinogen-mediated spleen tyrosine tinase (SYK) pathway in macrophages. It has been confirmed that a deficiency in ICAM-1 leads to the disruption of this crosstalk, a reduction in leukocyte infiltration and a delay in re-epithelialization in murine wounds 62-65. Concurrently, apoptotic neutrophils release Annexin A1 (ANXA1), which binds formyl peptide receptor 2 (FPR2/ALX) on macrophages to activate adenosine monophosphate-activated protein kinase (AMPK) signaling—a critical regulator of energy homeostasis. This interaction suppresses pro-inflammatory cytokine production and enhances the efferocytotic capacity through actin polymerization 66-68.

Efferocytosis induces epigenetic reprogramming in macrophages. Engulfment of apoptotic cells upregulates microRNA-21 (miR-21), which silences phosphatase and tensin homolog deleted on chromosome 10 (PTEN) and programmed cell death 4 (PDCD4) to enhance IL10 production and suppress the activities of pro-inflammatory cyclooxygenase-2 (COX-2) and inducible nitric oxide synthase (iNOS) 69. Clinically modified collagen gel (MCG) dressings have been demonstrated to accelerate diabetic wound healing by enhancing miR-21-c-Jun N-terminal kinase (JNK)-IL10 signaling, reducing MMP-9-mediated matrix degradation 70. The calcitonin gene-related peptide (CGRP) on sensory neurons binds to the receptor activity modifying protein 1 (RAMP1) receptors on macrophages to boost neutrophil efferocytosis and macrophage VEGF production. Impaired healing has been found in a diabetic neuropathy model due to CGRP depletion, while engineered CGRP nanoparticles can restore the phagocytotic function and promote angiogenesis 71,72.

During the proliferative phase, efferocytosis drives angiogenesis through multiple mechanisms. CXCR2 and CX3CR1 chemokine receptors coordinate endothelial cell migration and VEGF-mediated neovascularization, while a deficiency in CX3CR1 impairs capillary growth 73-75. Deferoxamine enhances vascularization by inducing the transcriptional activation of hypoxia-inducible factor 1 alpha (HIF-1α) to promote VEGF secretion. Functionalized apoptotic body nanovesicles containing deferoxamine (DFO-nABs) are used to target hypoxic endothelium via CX3CL1/CX3CR1 signaling, promoting angiogenesis and accelerating wound closure in preclinical models 76. MFG-E8 in synergy with VEGF can stabilize nascent vessels, and the sphingolipid mediator, S1P, enhances endothelial junction assembly through sphingosine-1-phosphate receptor 1 (S1PR1)-RAC1 signaling 77-79. The immunomodulator, FTY720 (fingolimod), is utilized to enhance angiogenesis by reducing VEGF-induced vascular permeability 79.

Efferocytosis facilitates re-epithelialization and matrix remodeling. Chaperone calreticulin (CRT), an “eat-me” signal on the endoplasmic reticulum, can bind to LDL-receptor-related protein (LRP) on fibroblasts to activate ERK/PI3K pathways to drive keratinocyte migration 80,81. Topical application of CRT, a typical “eat me” signal of efferocytosis, promotes macrophage migration, elevates the rate of reepithelialization, and enhances the rate and quality of wound healing in a diabetic murine model 82. S1P promotes epithelialization by inducing keratinocyte differentiation through the expression of STAT3-mediated filaggrin, while FTY720 enhances collagen deposition via the activation of the mothers against decapentaplegic homolog 2/3 (SMAD2/3) 83,84. Sonic Hedgehog (SHH), a member of the Hedgehog family and a secreted protein, binds to the patched receptor on target cells, activating the downstream transcription factor Gli 85. SHH promotes M2 polarization and augments macrophage efferocytosis by enhancing OXPHOS of macrophages, ultimately contributing to collagen deposition 86.

Recent evidence indicates that impaired efferocytosis may significantly contribute to chronic wound pathogenesis especially in diabetic condition. In a genetically-modified diabetic mouse model, refractory skin wounds demonstrated increased apoptosis, inhibited endothelial cell proliferation, diminished fibroblast function and decreased procollagen I mRNA expression 87. This pathologic condition is further prolonged by macrophage dysfunction since diabetic wounds demonstrated defective efferocytotic clearance of apoptotic cells. Khanna et al. have demonstrated that when impaired macrophage efferocytosis in diabetes, there will be an increase in the apoptotic cell burden and this leads to pro-inflammatory cascade with increased expression of TNF-α and IL-6 and a reduced level of IL10. This pro-inflammatory milieu further enhances inflammatory responses and greatly inhibits wound healing 60. In addition, hyperglycemia-induced advanced glycation end products (AGEs) may also lead to the inhibition of phagocytosis. These glycated proteins/lipids preferably accumulate in diabetic tissues. The level of glycated proteins/lipids in diabetic tissues is inversely correlated with the macrophage phagocytic capacity in peritoneal models 87-90. Mechanistically, AGEs bind to their receptor (RAGE) on M1 macrophages to lead to inhibition of phagocytosis. Treatment with anti-RAGE antibodies or soluble RAGEs (sRAGEs) restores macrophage function and improves neutrophil clearance and polarizes macrophages toward a pro-healing phenotype. Noteworthily, topical application of sRAGEs improves neo-vascularization and granulation tissue formation in diabetic wounds. Therefore, the AGE-RAGE signaling may be a potential target to heal diabetic wounds 91,92. The glycation induced inhibition also have an impact on some critical efferocytosis mediators like MFG-E8. In diabetic wound environment, the protein level of MFG-E8 was down regulated. Hyperglycemia induced glycation further leads to the impairment of its PS binding capacity. Both of these lead to the defect in apoptotic cell clearance. Recombinant MFG-E8 (rMFG-E8) supplementation improved macrophage efferocytosis, angiogenesis and resolved inflammation to accelerate diabetic ulcer healing 60. MicroRNA dysregulation also leads to diabetic wound pathology. Macrophage-derived miR-126 is a regulator that normally inhibits the expression of A disintegrin and metalloproteinase 9 (ADAM9) to maintain the efferocytosis efficiency. Under a high-glucose condition, miR-126 is suppressed, leading to overexpression of the ADAM9, which impairs apoptotic cardiomyocyte clearance, a phenomenon mirrored in human diabetic hearts characterized by impaired efferocytosis signaling. miR-126 overexpression has been demonstrated to enhance apoptotic cell clearance, and it could be a promising therapeutic approach for diabetes-impaired wound repair 93. Peroxisome proliferator-activated receptor gamma (PPAR-γ), a transcriptional regulator for the macrophage function, plays a dual role in wound healing. Injury normally induces upregulation of PPAR-γ to coordinate phagocytosis and inflammation resolution, while under a diabetic condition, sustained IL-1β expression can pronouncedly suppress the PPAR-γ activity. PPAR-γ knockout models display prolonged inflammation, reduced collagen deposition, and impaired angiogenesis due to accumulated apoptotic cells. Topical application of PPAR-γ agonists is demonstrated to accelerate healing in both normal and diabetic wounds by reactivating efferocytosis pathways 94-96. In addition to macrophages, efferocytosis by DCs is compromised in diabetic wounds via dysregulation of the solute carrier family 7 member 11(SLC7A11). This cysteine-glutamate transporter critically governs DCs-mediated apoptotic cell clearance, and pharmacological inhibition of the transporter restores the efferocytotic capacity and improves diabetic wound treatment outcomes 97.

**Figure 2:**
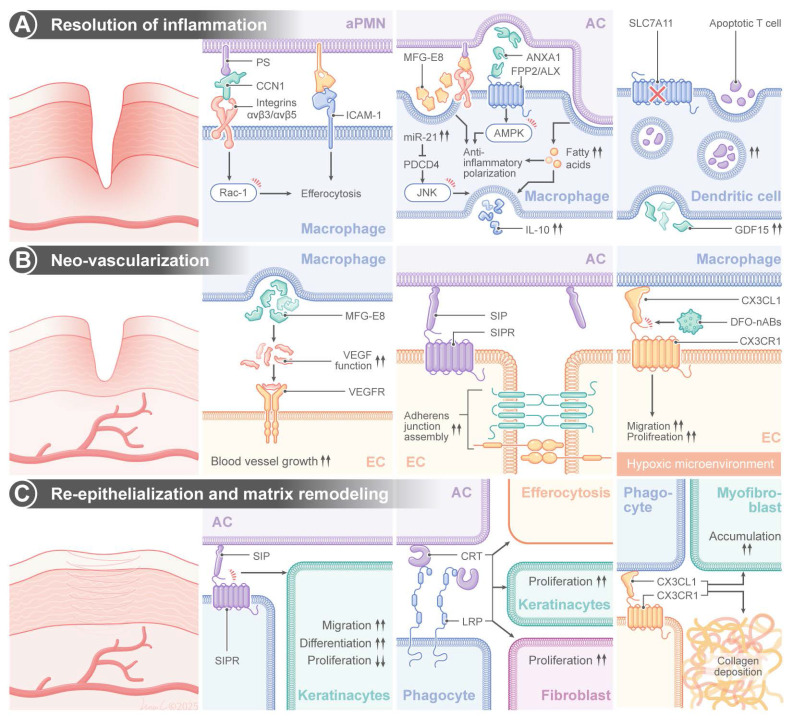
** Efferocytosis in cutaneous wound healing.** Efferocytosis play a critical role in regulating wound healing by (A) promoting the resolution of inflammation, (B) supporting neo-vascularization and (C) enhancing reepithelialization and matrix remodeling. CCN1: cellular communication network factor 1, ICAM-1: intracellular adhesion molecule-1, AC: apoptotic cell, aPMN: apoptotic polymorphonuclear neutrophils, MFG-E8: milk fat globule-epidermal growth factor 8, mi-R21: microRNA-21, ANXA1: Annexin A1, IL-10: interleukin-10, GDF15: growth differentiation factor-15, EC: endothelial cell, VEGF: vascular endothelial growth factor, VEGFR: vascular endothelial growth factor receptor, DFO-nABs: nanovesicles containing deferoxamine, CRT: calreticulin, LRP: LDL-receptor-related protein, JNK: c-Jun N-terminal kinase, AMPK: AMP-activated protein kinase.

Recent studies have indicated that bacterial pathogenesis plays a role in delaying chronic wounds. Staphylococcus aureus-derived vesicles (SAVs) inhibit efferocytosis by activating the toll-like receptor 2-myeloid differentiation primary response 88-p38 mitogen-activated protein kinase signaling (TLR2-MyD88-p38 MAPK) axis to promote cleavage of MerTK, a key efferocytosis receptor. The underlying mechanism is the culprit for the persistence of nonhealing SAV-infected wounds. Targeted inhibition of p38 MAPK is found to prevent MerTK degradation, restore efferocytosis and accelerate healing (**Figure 2**) 98.

### 3.2 Efferocytosis in liver injury

Liver injury represents a spectrum of pathological conditions characterized by hepatocyte death, sterile inflammation, and varying degrees of tissue remodeling. Efferocytosis plays an indispensable part in injured tissue repair by actively clearing apoptotic corpses. Beyond simple debris removal, it governs inflammation resolution, macrophage reprogramming, and fibrogenic signaling, dictating therapeutic outcomes of liver injury. Therefore, context-dependent cellular mediators often determine the effect of protection or pathogenesis induced by efferocytosis. Crucially, efferocytosis can be regulated to coordinate hepatic repair across injury paradigms, including hepatic ischemia-reperfusion injury (IRI), metabolic/alcoholic damage, and toxic insults, by modulating inflammation and promoting tissue regeneration.

In models of hepatic IRI, multiple molecular pathways for the promotion of apoptotic cell clearance have been identified. A deficiency in CX3CR1 amplifies C-C chemokine receptor type 1/5 (CCR1/5)-mediated macrophage migration and liver X receptor α (LXRα)-mediated MerTK-dependent efferocytosis, thus repopulating the Kupffer cell (KC) niche via reparative macrophages reprogramming and facilitating IRI resolution. Pharmacological inhibition of CX3CR1 via an antagonist, AZD8797, attenuates hepatic IRI severity, confirming it could be a therapeutic target 99. In addition, Ming *et al*. have identified T-cell immunoglobulin and mucin domain-containing protein 4 **(**TIM-4) as a critical regulator for efferocytosis and KC homeostasis. TIM-4, a PS receptor, promotes KC efferocytosis by suppressing pro-inflammatory TNF-α production and upregulating the secretion of anti-inflammatory IL10 upon Toll-like receptor activation. A deficiency in TIM-4 compromises the efferocytosis capacity of KCs, leading to aggravated hepatocellular injury in the early phase and impaired resolution of inflammation in the later phase 100. During the hepatic IRI inflammation resolution phase, TREM2 in pro-resolution macrophages facilitates the recognition and internalization of ccumulated apoptotic cells via COX2/PGE2-mediated RAC1 activation. Efferocytosis drives phenotypic transition of monocyte-derived macrophages into reparative CX3CR1^+^Ly6C^lo^, which orchestrates inflammation resolution and promotes liver regeneration 101. It has been confirmed that Resolvin D1 (RvD1), a pro-resolving lipid mediator, confers protection against hepatic IRI, and the protective effect is ascribed to enhancements in M2 polarization and efferocytosis via lipoxin A4 receptor/formyl peptide receptor 2 (ALX/FPR2) activation 102. Interestingly, for the first time, the anti-inflammatory and pro-regenerative effects of netrin-1, a neuroimmune guidance cue, on hepatic IRI are revealed, supporting the role of neuroimmune molecules in a non-neural system. Netrin-1 may serve as a therapeutic target for hepatic IRI. Attenuating injury and promoting liver regeneration can be achieved through exogenous administration of netrin-1 or modulation of its signaling pathway 103.

In chronic liver injury models, including alcohol-associated liver disease (ALD) and metabolic dysfunction-associated steatohepatitis (MASH), efferocytosis also plays a pivotal role. In alcohol-induced liver injury models, RvD1 promotes macrophage efferocytosis of apoptotic hepatocytes and enhanced clearance of dead hepatocytes mitigates hepatic damage, thus RvD1 could be used as a therapeutic adjuvant to support liver regeneration through anti-inflammatory repair 104. The hepatocyte-derived S1P signal via S1PR1 upregulates the expression of TREM2, a phagocytotic receptor on infiltrated macrophages, to achieve efficient efferocytosis of lipid-laden apoptotic hepatocytes, maintain tissue immune homeostasis and prevent MASH development. Nevertheless, prolonged hypernutrition triggers TNF/IL-1β-mediated ADAM17-dependent proteolytic cleavage of TREM2, thus depleting functional soluble TREM2 (sTREM2). This loss impairs efferocytosis, leading to aberrant accumulation of apoptotic debris, which contributes to chronic liver inflammation and MASH progression 105,106. Furthermore, bioinformatics and machine learning analysis of human liver transcriptomes reveal that efferocytosis impairment is induced through upregulated TREM2 and downregulated TIM-4, leading to polarization towards an M1 phenotype and reduction in the M2 population, which drive unresolved inflammation. Notably, TREM2/TIM-4 signatures have been used to precisely diagnose MASH, suggesting their clinical use for early diagnosis and stratification of the disease 107. To treat toxic insults such as acetaminophen-induced injury, natural antibodies (NAbs) are used to drive the phagocytosis of necrotic hepatocytes via the Fc gamma receptor (FcγR) and integrin alpha M (CD11b) to fuel liver regeneration. NAbs supplementation enhances the efferocytotic capacity and increases the hepatic density, providing therapeutic benefits 108.

The identity and phenotype of efferocytotic macrophage subsets are critical for the outcome of therapeutic modalities for liver injury. Macrophage efferocytosis of hepatocyte debris induces the formation of a restorative matrix-degrading Ly6C^lo^ phenotype with activated ERK signaling, which promotes the resolution of tissue fibrosis. Inducing efferocytosis using liposomes expands the population of this restorative macrophage phenotype *in vivo* to accelerate liver fibrosis regression 109. Proteomic analysis confirms that Ly6C^lo^CX3CR1^hi^ macrophages display an enriched level of several reparative efferocytosis-related proteins, including arachidonate 15-lipoxygenase (ALOX15), a marker associated with inflammation resolution and tissue repair. The macrophage subpopulation directly accelerates hepatocyte proliferation via the secretion of hepatocyte growth factors (HGFs). Moreover, selective depletion of this subpopulation hinders liver regeneration, confirming the essential role of this subpopulation in efferocytosis-driven liver repair 110,111. In addition, both *in vitro* and in acetaminophen (APAP)-treated mice, the secretory leucocyte protease inhibitor (SLPI), a microenvironmental mediator, has been shown to reprogram myeloid cells for resolution responses by inducing the formation of a pro-restorative MerTK^+^HLA-DR^high^ phenotype. This phenotype displays enhanced efferocytosis of apoptotic neutrophils to promote liver regeneration 112. The STAT3-IL10-IL6 autocrine-paracrine pathway has been identified as a positive regulator of macrophage efferocytosis and phenotypic conversion, and this pathway plays a critical role in disposal of apoptotic bodies and tissue repair. Disruption of this axis results in defective clearance of apoptotic cells, impaired macrophage reprogramming, and delayed resolution of liver injury, while IL-6 treatment restores the phagocytic function and improves regenerative outcomes 113. Mechanical cues also modulate these activities. Upregulation of the mechanosensitive ion channel Piezo1 in macrophages during hepatic fibrosis enhances their stiffness-dependent efferocytosis. Consistently, macrophages without Piezo1 exhibit sustained inflammation and impaired spontaneous resolution of liver fibrosis, whereas pharmacological activation (e.g. Yoda1) of Piezo1 alleviates fibrotic progression 114.

Efferocytosis-driven tissue remodeling is not limited to hepatocyte contexts. After bile duct decompression, apoptotic cholangiocytes are efficiently cleared by recruited macrophages via efferocytosis, and these macrophages are reprogrammed into a fibrolytic phenotype. The efferocytosis-induced transition is marked with robust upregulation of matrix metalloproteinases (MMPs), which mediate ECM degradation. Concurrently, the removal of dying cholangiocytes suppresses profibrotic signaling, promoting coordinated inflammation resolution and tissue remodeling 115.

Impaired efferocytosis exacerbates inflammation and fibrosis after liver injury. Aging exacerbates hepatic IRI by impairing MerTK-mediated macrophage efferocytosis. Excessively produced ROS drives ADAM17-mediated MerTK cleavage, leading to the accumulation of apoptotic hepatocytes. Subsequently, DNA from dying hepatocytes activates stimulator of interferon genes (STING) signaling in macrophages, contributing to sustained inflammation and tissue injury in aged livers 116. Besides, it has been demonstrated that the NOD-like receptor protein 3 (NLRP3) inflammasome hinders liver regeneration and inhibits hepatocyte proliferation through impairing MerTK-mediated efferocytosis by macrophages and preventing subsequent polarization of macrophages toward a pro-reparative Ly6C^lo^ phenotype. Pharmacological inhibition of NLRP3 via MCC950 effectively restores the efferocytotic capacity and promotes liver regeneration after 70% partial hepatectomy in mice fed with a high-fat diet 117. Similar defects associated with MerTK are found in ALD. A deficiency in gp91^phox^, a catalytic subunit in NOX2, impairs hepatic efferocytosis by macrophages, which is attributed to downregulated expression of key phagocytotic receptors including MerTK and TIM-4. The deficiency in the catalytic subunit results in the accumulation of apoptotic cells and prevents programming of macrophages from a pro-inflammatory to a tissue-restorative phenotype, elevating the severity of ALD and inhibiting hepatic tissue repair 118. Moreover, in patients with alcoholic hepatitis (AH), neutrophil-derived neutrophil extracellular traps (NETs) exacerbate liver injury and resist efferocytosis by macrophages that are chronically exposed to alcohol. The persistence of NETs induces macrophages pro-inflammatory differentiation, sustaining tissue damage. Transcriptomic analysis has identified that an elevated level of CD47 expression on low-density neutrophils (LDNs) from AH patients is coupled with PS downregulation, jointly impairing neutrophil clearance. The neutrophil-macrophage dysregulation intensifies NET-driven inflammation and hinders liver repair 119. In MASH, necroptotic hepatocytes displayed an upregulated level of CD47, while liver macrophages exhibit an increased level of SIRPα expression, collectively impairing efferocytosis. Blocking the CD47-SIRPα axis restores efferocytosis of necroptotic hepatocytes by macrophages and ameliorates liver fibrosis 120.

Macrophages with a deficiency in myeloid Niemann-Pick C1 (NPC1), a lysosomal cholesterol transport protein, exhibit a diminished capacity in the degradation of apoptotic cargoes like mitochondrial DNA, leading to excessive DAMPs accumulation, aggravated hepatic inflammation, and STING/NFκB pathway activation. Inefficient efferocytosis hinders macrophage reprogramming toward a reparative phenotype and intensifies liver fibrosis, thereby impairing tissue regeneration 121. Miyazaki *et al*. have demonstrated that the absence of fatty acid binding protein 7 (FABP7), exclusively localized in KCs, impairs efferocytosis primarily due to downregulated expression of the scavenger receptor CD36. The defect leads to deteriorated liver injury and a reduction in the levels of TNF-α, monocyte chemoattractant protein-1 (MCP-1), and TGF-β1. Consequently, FABP7-deficient mice exhibit insufficient fibrogenic responses during chronic liver injury 122.

Emerging evidence has suggested that under a pathological condition, maladaptive efferocytosis may paradoxically drive fibrosis through the activation of hepatic stellate cells (HSCs). Macrophages typically reprogram toward a pro-resolving phenotype upon uptake of apoptotic cells, while HSCs, the key fibrogenic cells in the liver, exhibit profibrotic transformation after a distinct response to engulfed apoptotic materials. Early in the injury response, CCN1 facilitates efferocytosis by liver macrophages through bridging PS on apoptotic cells and integrin αvβ3 on phagocytes, which triggers the polarization of macrophages towards an M2-like phenotype that expresses fibrogenic TGF-β1. Besides, efferocytosis has been demonstrated to induce HSC transdifferentiation into myofibroblast-like cells, contributing to fibrosis development in chronic liver injury 123. Canbay* et al.* have demonstrated that in bile duct-ligated mice, KC efferocytosis of apoptotic hepatocytes enhances Fas Ligand (FasL)/TNF-α expression, amplifying a feed-forward loop of hepatocyte apoptosis. This process drives hepatic fibrosis via cytokine storm production, neutrophil infiltration, and HSC activation. Inhibition of KCs or hepatocyte apoptosis attenuates these effects, suggesting there is a pathogenic link between efferocytosis and liver injury 124. Another study has indicated that efferocytosis of apoptotic bodies (ABs) by HSCs directly triggers liver fibrosis through upregulation of procollagen α1 and TGF-β1. The upregulation of procollagen α1 may activate nicotinamide adenine dinucleotide phosphate (NADPH) oxidase, resulting in excess superoxide production. Therefore, efferocytosis could be as a key mechanism for converting physiological removal of debris into pathological fibrogenesis 125.

Pathogen-associated or metabolite-induced ABs exacerbate the maladaptive response. After ABs from hepatocytes are exposed to the alcohol metabolite, acetaldehyde, or human immunodeficiency virus (HIV), these Abs are engulfed by HSCs via Axl with the help of two bridging molecules, Gas 6 and protein S. This efferocytosis process triggers ROS-dependent JNK-ERK1/2 and janus kinase** (**JAK)-STAT3 pathways, leading to enhanced expression of profibrotic genes and HSC activation 126. Muhanna *et al*. have uncovered a novel fibrogenesis pathway. HSCs engulf disease-associated lymphocytes, particularly CD8^+^ T cells, leading to their activation. The activation is mediated via ICAM-1/integrin αV interaction through RAC1 and cell division control protein 42 (CDC42) signaling pathways. Efferocytosis of lymphocytes enhances α-SMA expression and TGF-β signaling in HSCs, aggravating injury and strengthening fibrosis while suppressing regeneration in patients with chronic viral hepatitis 127.

**Figure 3:**
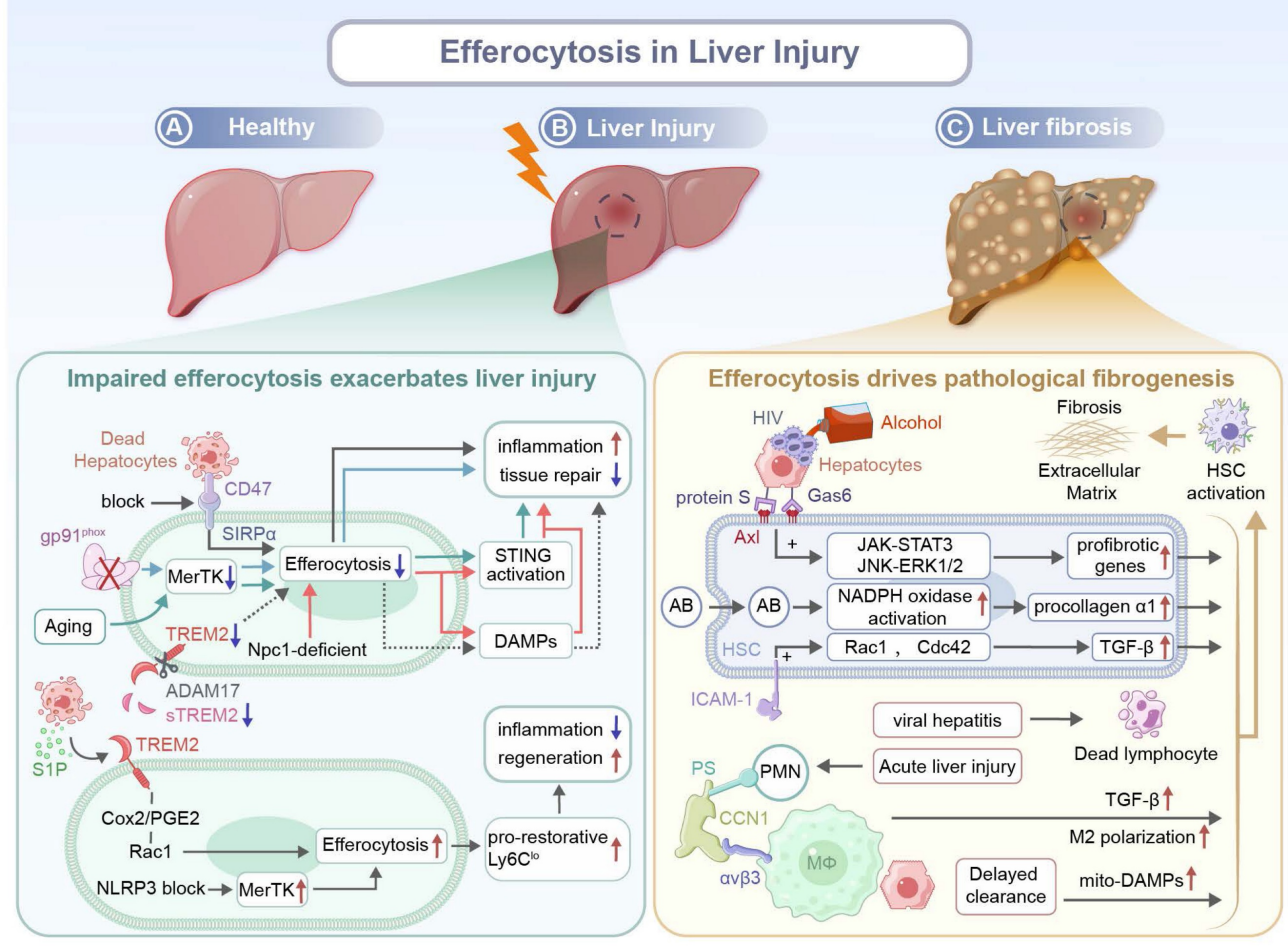
** Roles of efferocytosis in liver injury and fibrosis.** (A) In a healthy liver, apoptotic cell clearance maintains immune tolerance and tissue integrity. (B) During liver injury, impaired efferocytosis caused by CD47 blockade, MerTK deficiency, TREM2 downregulation, aging, or Npc1 mutation leads to reduced uptake of apoptotic cells, enhanced STING activation, DAMP release, persistent inflammation, and impeded repair. In contrast, signals, such as S1P, Cox2/PGE2, and Rac1, promote the generation of pro-restorative Ly6C^lo^ macrophages and tissue regeneration. CD47: cluster of differentiation 47, MerTK: Mer receptor tyrosine kinase, TREM2: triggering receptor expressed on myeloid cells 2, Npc1: Niemann-Pick type C1, STING: stimulator of interferon genes, DAMPs: damage-associated molecular patterns, S1P: sphingosine-1-phosphate, Cox2: Cyclooxygenase-2, PGE2: Prostaglandin E2, Rac1: Ras-related C3 botulinum toxin substrate 1, Ly6C^lo^: Lymphocyte antigen 6 complex, locus C low. (C) During liver fibrosis, efferocytosis of apoptotic hepatocytes induced by alcohol, viral hepatitis, or HIV activates hepatic stellate cells (HSCs) through Axl/Protein S/Gas6 signaling. The signaling pathway triggers the activation of JAK-STAT3, JNK-ERK1/2, and NADPH oxidase pathways, resulting in TGF-β release, profibrotic gene induction, and collagen deposition. Additional mechanisms, including ICAM-1-mediated interactions, CCN1/αvβ3 signaling, M2 polarization, and delayed apoptotic cell clearance, promotes extracellular matrix accumulation and fibrogenesis. HIV: Human immunodeficiency virus, HSCs: Hepatic stellate cells, Axl: AXL receptor tyrosine kinase, Gas6: Growth arrest-specific 6, JAK-STAT3: Janus kinase-signal transducer and activator of transcription 3, JNK-ERK1/2: c-Jun N-terminal kinase-extracellular signal-regulated kinase 1/2, NADPH: Nicotinamide adenine dinucleotide phosphate, Cdc42: Cell division cycle 42, TGF-β: Transforming growth factor-beta, ICAM-1: Intercellular adhesion molecule 1, PS: Phosphatidylserine, PMN: Polymorphonuclear neutrophil, CCN1: Cellular communication network factor 1, αvβ3: Integrin alpha-v beta-3, MΦ: Macrophage, mito-DAMPs: Mitochondrial damage-associated molecular patterns.

Importantly, delayed or inefficient clearance of apoptotic hepatocytes by professional phagocytes such as macrophages may indirectly promote HSC activation. The prolonged presence of uncleared apoptotic hepatocytes induces continuous release of mitochondria-derived DAMPs, which directly activate HSCs to drive progression of liver fibrosis independent of classical inflammation pathways. The experimental data has supported that both defective and misdirected efferocytosis leads to intensified fibrosis (**Figure 3**) 128.

### 3.3 Efferocytosis in myocardial repair

Myocardial injury, particularly myocardial infarction (MI), initiates a complex repair process characterized by dynamic inflammatory resolution, cardiomyocyte repopulation, and fibrotic remodeling. The poor regenerative ability of cardiomyocytes and the inflammatory milieu have been identified as critical barriers for ventricular remodeling and heart regeneration, and cardiac healing becomes more challenging than superficial wound healing 129-131.

Post-MI cardiac repair progresses through three overlapping phases: inflammation, proliferation, and maturation, and the repair process is mediated by dynamic interactions among neutrophils, macrophages, fibroblasts, endothelial cells, cardiomyocytes, and stem cells through paracrine signaling and cell-cell communication 132,133. After IRI, necrotic cardiomyocytes release DAMPs to trigger rapid neutrophil infiltration. Effective clearance of these neutrophils in the injury site is critical to prevent secondary necrosis and prolonged inflammation 134,135. Efferocytosis, phagocytic clearance of apoptotic cells including cardiomyocytes and neutrophils, serves as a pivotal mechanism to resolve inflammation and coordinate tissue regeneration in the infarcted heart. Dysregulated efferocytosis, however, drives pathological fibrosis and chronic inflammation 132.

Central to this process is MerTK, a key efferocytosis receptor with a context-dependent role in disease pathogenesis. While genomic studies have revealed the protective or detrimental effect of MerTK during pathogenesis, its specific role in MI repair has been recently clarified 136. Wan *et al*. have demonstrated dynamic upregulation of MerTK in the left ventricular ischemic zone peaks on day 7 post-MI, thus macrophages can efficiently clear apoptotic cardiomyocytes. This MerTK-dependent efferocytosis process suppresses secondary necrosis, facilitates inflammation resolution, and promotes tissue regeneration, supporting there is a direct mechanistic link between apoptotic cell clearance and inflammatory-to-reparative transition 137. The reparative potential of MerTK is demonstrated in an ischemia-reperfusion model. Cleavage-resistant MertkCR reduces the production of soluble MerTK (solMER), enhances phagocytosis, and improves therapeutic outcomes. Resident major histocompatibility complex class II/low expression C-C chemokine receptor type 2 (MHCIILOCCR2) macrophages drive this repair process through TGF-β secretion, while recruited monocytes intensify MerTK cleavage via proteolytic inactivation. Therapeutic CCR2 blockade preserves MerTK integrity, and it could be a promising strategy to mitigate reperfusion injury 138. Interestingly, cardiomyocyte debris impairs efferocytosis by inducing MerTK shedding through cardiomyocyte smacrophage crosstalk. Despite macrophage chemotaxis and apoptotic CM binding, poor engulfment of apoptotic cells is observed due to cardiomyocytes-triggered proteolysis of the extracellular domain of MerTK. Genetic stabilization of the MerTK cleavage site can partially restore the phagocytic capacity, suggesting MerTK may be a critical factor for cardiac injury resolution 139. In contrast to a pro-repair role of MerTK, injured cardiomyocytes upregulate CD47, a “don't eat me” signal. The signal binds to SIRPα on macrophages to suppress efferocytosis. The interaction between CMs and macrophages perpetuates inflammation and exacerbates injury, while acute CD47 blockade leads to improved infarct resolution and functional recovery. The balance between “eat me” (e.g., MerTK) and “don't eat me” (e.g., CD47) signals has a critical impact on repair outcomes, and they could be therapeutic targets for efferocytosis regulation 140.

Emerging evidence supports that cardiac debris clearance can be achieved by other cell types in addition to macrophages. Myofibroblasts exhibit a high phagocytic capacity through MFG-E8-dependent mechanisms. They secrete anti-inflammatory TGF-β, while inhibiting IL-6 production to create a pro-reparative microenvironment 141. In addition, TIM-4^+^ resident macrophages uniquely mediate PS recognition and apoptotic cell clearance through the PS receptor. The depletion of TIM-4^+^ resident macrophages impairs the cardiac function although compensatory macrophages are recruited to replace TIM-4^+^ resident macrophages, suggesting an indispensable role of TIM-4^+^ resident macrophages in inflammation resolution 142.

Efferocytosis is significantly impacted by cellular metabolism. In diabetic cardiomyopathy, hyperglycemia disrupts the miR-126/ADAM9/MerTK axis, impairing macrophage efferocytosis and impeding myocardial repair 93. By contrast, TREM2^+^ macrophages can perform efferocytosis after metabolic adaptation through the SYK-SMAD4 pathway. The downregulation of the engulfment-induced solute carrier family 25 member 53 (SLC25A53) disrupts mitochondrial NAD^+^ transport, enhancing itaconate production to suppress cardiomyocyte apoptosis and promote fibroblast proliferation. The metabolic shift suggests there is a tight integration between the phagocytic function and cellular metabolism during a repair process 143. Another unique metabolic pathway associated with efferocytosis is recently revealed. Neonatal cardiac injury activates C1q^+^TLF^+^ macrophages through MerTK-dependent efferocytosis, and arachidonic acid metabolism is redirected to thromboxane A2 (TXA2). TXA2 binding to cardiomyocyte G-protein-coupled thromboxaneprostanoid receptors triggers glycolytic reprogramming and regenerative repair mediated by Yes-associated protein 1/transcriptional co-activator with PDZ-binding motif (YAP/TAZ) 144.

Key molecular regulators have been gradually identified from different experimental models. Macrophage-derived legumain (LGMN) enhances LC3-II-dependent phagosome formation and activates calcium signaling to accelerate apoptotic cell clearance, reduce IL-1β/TNF-α release and improve post-MI remodeling 145; TGF-β-activated SMAD3 promotes MFGE8-mediated efferocytosis and activates PPAR-γ/δ anti-inflammatory signaling, while a deficiency in SMAD3 leads to ineffective clearance of apoptotic cells and hindered remodeling 146; Neutrophil-secreted neutrophil gelatinase-associated lipocalin (NGAL) polarizes macrophages toward an M2 phenotype with elevated MerTK expression and an enhanced efferocytotic capacity 147; Galectin-3^hi^CD206^+^ macrophage-derived osteopontin (OPN) enhances efferocytosis through IL10-STAT3 signaling, which is critical for reparative fibrosis and ventricular stability 148; Neogenin 1 (NEO1) can maintain the efferocytotic capacity by suppressing JAK1-STAT1-mediated proinflammatory polarization 149.

Profiling the cellular landscape during cardiac repair reveals functional specialization of macrophage subsets. Embryo-derived MHC-II^hi^ macrophages maintain tissue homeostasis through constitutive efferocytosis, while bone marrow-derived MHC-II^lo^ macrophages play a vital role in post-MI necrotic debris clearance. Therapeutic targeting of these distinct subpopulations could achieve optimized inflammation resolution and tissue regeneration 150. Notably, CD36 on the recruited Ly6c^hi^ monocytes initiates the nuclear receptor subfamily 4 group A member (NR4A1)-MerTK signaling cascade that drives macrophage maturation and repair programming 151.

In contrast to the pro-repair mechanisms, pathological matrix remodeling creates barriers for efferocytosis. The accumulation of high molecular-weight hyaluronan (HAHMW) in infarcted hearts physically impedes phagocytosis by macrophages through CD44-independent mechanisms, particularly in naive (M0) and pro-inflammatory (M1) macrophage subsets. The suppression of efferocytosis delays inflammation resolution, sustains IL-17/IP-10 signaling, and promotes maladaptive remodeling through persistent autoantigen exposure. Therefore, manipulation of biophysical properties of HA, or modulation of the ECM composition could improve the phagocytic efficiency 152.

**Figure 4:**
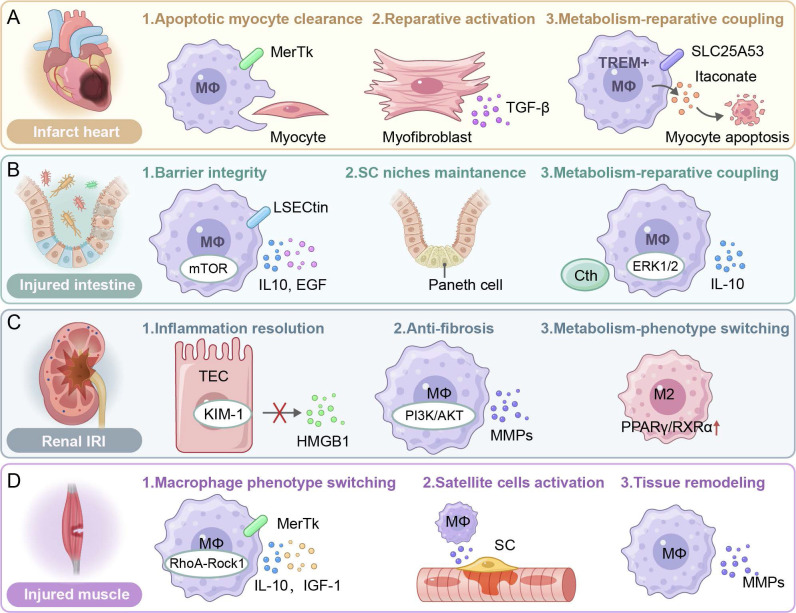
**Roles of efferocytosis in the injury of the heart, intestine, kidney and muscle.** (A) Infarct heart. Apoptotic myocyte clearance via MerTk receptor signaling; reparative activation of myofibroblasts through TGF-β secretion; metabolic coupling via SLC25A5353-mediated transport or TREM⁺ macrophage-derived itaconate. (B) Injured intestine. The barrier integrity is maintained via LSECtin/mTOR and IL-10/EGF pathways; the stem cell niche supports through Paneth cell interactions; metabolism-repair coupling is mediated by ERK1/2/IL-10 and Cth. (C) Renal IRI. Inflammation resolution via the KIM-1/TEC axis and HMGB1 modulation; anti-fibrotic effects through PI3K/AKT/MMPs signaling; M2 polarization driven by PPARγ/RXRα transcriptional activation. (D) Injured muscle. Phenotype switch of macrophages (MΦs) via RhoA-Rock1 mechanotransduction; satellite cell (SC) activation by IL-10/IGF-1 paracrine signaling; tissue remodeling facilitated by MMPs and recurrent MerTk-dependent clearance. SLC25A53: Solute carrier family 25, member 53. LSECtin: Lymphatic endothelial cell lectin, mTOR: Mammalian target of rapamycin, IL-10: Interleukin-10, EGF: Epidermal growth factor, Cth: Cystathionine gamma-lyase, ERK1/2: Extracellular signal-regulated kinase 1/2. TEC: Tubular epithelial cell, KIM-1: Kidney injury molecule-1, HMGB1: High mobility group box 1, PI3K: Phosphoinositide 3-kinase, AKT: Protein kinase B, MMPs: Matrix metalloproteinases, PPARγ: Peroxisome proliferator-activated receptor gamma, RXRα: Retinoid X receptor alpha. RhoA: Ras homolog family member A, Rock1: Rho-associated kinase 1, IGF-1: Insulin-like growth factor-1, SC: Satellite cell.

Paradoxically, excessive activation of efferocytosis may be detrimental in the chronic injury context. A deficiency in the G protein-coupled receptor, class C, group 5, member B (GPRC5B) enhances macrophage efferocytosis through blocking E prostanoid receptor 2 (EP2 receptor) signaling and reducing cyclic adenosine monophosphate (cAMP)-mediated anti-inflammatory responses. While macrophage efferocytosis is beneficial for bacterial clearance to prevent infections, it induces imbalanced macrophage polarization in MI, enhancing myeloid cell recruitment but exacerbating inflammation and fibrosis. These findings suggest the context-dependent role of efferocytosis. It is essential for acute injury resolution, while it may be disruptive during chronic tissue repair when it is improperly regulated (**Figure 4**A) 153.

### 3.4 Efferocytosis in intestinal injury

The intestinal mucosa is characterized by a high proliferative capacity. It is a dynamic interface, and epithelial homeostasis is maintained through timely efferocytosis after it is exposed to various types of injury stress. Recent evidence has suggested that efferocytosis is pivotal in repairing diverse intestinal injury types, including the inflammatory bowel disease (IBD), chemically induced colitis, intestinal IRI, and radiation-induced enteropathy. Efficient efferocytosis not only prevents secondary necrosis and mitigates the release of pro-inflammatory intracellular contents but also actively shapes the resolution of inflammation and promotes tissue regeneration.

In murine models of dextran sodium sulfate (DSS)-induced colitis, the G2A deficient mice exhibit aggravated colitis, evidenced by the heightened disease activity, colon shortening and histopathological deterioration. In the G2A deficient mice, fewer CD4^+^ lymphocytes are recruited to the inflamed colon and more TNFα^+^ that is secreted by pro-inflammatory monocytes in an eosinophil-dependent manner is released, leading to impaired efferocytosis 154.

More recently, growing evidence has suggested that the effects of efferocytosis on ameliorating intestinal inflammation are pronounced during the recognition phase. It has been shown that the C-type lectin receptor, LSECtin, on macrophages contributes to the engulfment of apoptotic cells by macrophages. Additionally, LSECtin induces the expression of anti-inflammatory/tissue repair factors in an engulfment-dependent manner, such as TGF-β, VEGF, and heparin-binding epidermal growth factor-like growth factor (HBEGF), which in turn stimulates epithelial cell proliferation and promotes intestinal epithelium regeneration 155. Furthermore, apoptotic cells modulate intracellular signaling dynamics by enhancing the interaction between LSECtin and mammalian target of rapamycin (mTOR), thereby strengthening LSECtin-mediated activation of the mammalian target of rapamycin complex 1 (mTORC1) in macrophages. Subsequently, the heightened mTORC1 activity orchestrates transcriptional upregulation of anti-inflammatory/tissue repair factors, including IL10 and HBEGF. These mediators expedite intestinal regeneration by synchronizing epithelial restitution with inflammation resolution to restore tissue homeostasis 156. In both DSS- and 2,4,6 trinitrobenzene sulfonic acid (TNBS)-induced colitis models, the colons of animals receiving recombinant human MFG-E8 (rhMFG-E8) exhibit significant attenuation of neutrophil infiltration, downregulation of pro-inflammatory mediators, and a decrease in the apoptotic cell counts. These findings collectively demonstrate that rhMFG-E8 ameliorates DSS- and TNBS-induced colitis and promotes regeneration, supporting its potential as a therapeutic agent for IBD 157. Ravichandran et al. have found that BAI1 deficiency augments colitis severity by impairing efferocytosis after acute intestinal injury. Conversely, transgenetically boosting the BAI1 expression improves apoptotic cell clearance via ELMO1/DOCK1/RAC1 signaling, attenuates inflammation, and facilitates epithelial repair in murine DSS-induced colitis models. Critically, intestinal epithelial-specific BAI1 augmentation is sufficient in mitigating the pathological burden and accelerating mucosal regeneration 158. The absence of COX-2 in macrophage aggravates IBD progression by disrupting reparative efferocytosis. Meriwether *et al*. have demonstrated that COX-2 bolsters the macrophage phagocytotic capacity and triggers phenotypic reprogramming, enhancing intestinal epithelial repair. COX-2 deletion impairs the engulfment of apoptotic neutrophils, which may be due to a reduction in the binding affinity, while macrophage polarization is skewed away from an efferocytosis-competent transcriptional profile. Mechanistically, COX-2 regulates the synthesis of efferocytosis-associated lipid mediators, including prostaglandin I2 (PGI2), PGE2, lipoxin A4 (LXA4), and 15-Deoxy-Δ12,14-prostaglandin J2 (15d-PGJ2), and these lipid mediators can also impact secondary efferocytosis 159,160. Additionally, the ablation of CD300f, a phosphatidylserine receptor that recognizes apoptotic intestinal epithelial cells (IECs), exacerbates the pathogenesis of IBD. In murine colitis models, CD300f on DCs as professional phagocytes is essential for maintaining gut immune homeostasis. The CD300f-deficient mice exhibit impaired DSS resolution although these mice have hyperactive phagocytotic activity. Paradoxically, CD300f^-/-^ DCs are induced to overexpress TNF-α, which subsequently drives aberrant IFN-γ production by colonic T cells and perpetuates gut inflammation 161.

In the internalization phase of efferocytosis, ChemR23, a pro-resolving G protein-coupled receptor (GPCR) overexpressed on neutrophils and macrophages in therapy-refractory IBD, drives pathological neutrophil accumulation, leading to failure in inflammation resolution. An agonistic anti-ChemR23 monoclonal antibody (mAb) can bind to overexpressed ChemR23, mimicking resolvin E1 signals through ERK/Akt pathways to promote macrophage efferocytosis of apoptotic neutrophils, accelerate recovery from acute inflammation, and prevent fibrosis 162.

In the digestion phase, the activation of the homodimeric erythropoietin receptor (EPOR) on macrophages creates an anti-inflammatory microenvironment for tissue regeneration by enhancing efferocytosis, which may be dependent on LAP. Besides, EPOR activation remarkably promotes the production of anti-inflammatory/tissue repair factors including TGF-β, MRC1, chitinase-like protein 3 (YM1), PPAR-γ, MerTK and CD36, which are essential for apoptotic cell engulfment. ARA290, an erythropoietin (EPO) derivative, has been identified to orchestrate the restoration of the intestinal barrier function through upregulation of the expression of Mucin 2 (MUC2), a goblet cell mucin and augmentation of mucus secretion, partly by promoting the production of TGF-β 163. Another study suggests that nuclear receptor binding factor 2 (NRBF2), a regulatory subunit of the autophagy-related PI3KC3 complex, is critical in promoting efferocytosis and resolving intestinal inflammation 164. Additionally, LXR-deficient mice are susceptible to colitis induction. The LXR expression is reduced in the colonic tissues of IBD patients. Pharmacologic activation of LXR in colonocytes suppresses NF-κB and MAP kinase signaling via ATP-binding cassette transporter subfamily A member 1 (ABCA1), reducing pro-inflammatory chemokines (e.g. IL-8, C-C motif chemokine ligand 28 (CCL)-28) and promoting resolution of inflammation. The anti-inflammatory milieu may support efficient efferocytosis by inhibiting excessive immune activation, thereby facilitating tissue repair 165,166. Besides, PPAR-γ activation effectively ameliorates IBD progression by orchestrating the regulation of epithelial-mesenchymal transition (EMT), AGE/RAGE signaling, and cellular senescence pathways 167.

The SuperMApo (SUPERnatant collected from Macrophage APOptotic cell culture) is enriched in pro-resolving factors produced by macrophages after efferocytosis, including several chemokines (CCL5, CXCL2, and CCL22) and cytokines (IL-1RA, IL10, and TGF-β). The SuperMApo hinders progression of IBD by mitigating intestinal inflammation and promoting mucosal healing. Specifically, the SuperMApo suppresses inflammatory cell infiltration into colitis lesions. It also triggers the activation of wound healing in fibroblasts and IECs, which is supported by their enhanced proliferative and migratory properties 168.

Intestinal IRI is an acute injury characterized by massive epithelial apoptosis in the intestinal mucosa and its associated lymphoid tissues followed by an intense sterile inflammatory response. Massive epithelial apoptosis elevates the burden for efferocytosis, while enhanced efferocytosis through MFG-E8 and metabolic enzymes becomes critical for inflammatory resolution 169. In an established animal model of intestinal IRI, the intestinal level of MFG-E8 is significantly reduced. Enhancing apoptotic cell clearance by supplementing rmMFG-E8 suppresses systemic inflammatory responses, attenuates intestinal injury, and promotes VEGF-mediated tissue repair 170,171. Cystathionine gamma-lyase (Cth) is an enzyme that catalyzes the conversion of cystathionine into cysteine, α-ketobutyric and ammonia, which are intermediate metabolites involved in H_2_S production. Cth has been shown to affect the intestinal function by promoting the synthesis of H_2_S to treat colitis 172,173. Moreover, Cth activates macrophage efferocytosis via the ERK1/2 signaling pathway through the production of H_2_S both *in vivo* and *in vitro*, which in turn regulates intestinal inflammation and promotes intestinal mucosal repair. Pharmacological inhibition of Cth or ERK1/2 impairs mucosal repair, while agonists for Cth or ERK1/2 can accelerate the mucosal recovery. Clinically, the Cth level is positively correlated with the efferocytosis activity and the level of inflammation resolution, and a higher Cth level results in faster barrier recovery in patients 174.

Interestingly, using irradiation mice models and intestinal organoids, Shankman *et al*. have discovered a novel role of Paneth cells in phagocytic clearance of apoptotic IECs. Paneth cells residing in intestinal crypts can act as phagocytes, and enhancing their efferocytosis may offer novel therapeutic strategies to alleviate mucosal injury in the contexts of chemotherapy or chronic IBD because unresolved apoptosis in these contexts exacerbates tissue damage, impairs the innate immunity, and disrupts the stem cell niche in the crypt (**Figure 4**B) 175.

### 3.5 Efferocytosis in kidney injury

Renal tissues injured by ischemic or toxic insults display acute tubular epithelial cell (TECs) degeneration, basement membrane disruption, interstitial inflammation, and occasional glomerular mesangial expansion. In mild acute kidney injury (AKI), surviving TECs dedifferentiate, migrate, and proliferate to repopulate the tubular epithelium, however, severe injury leads to persistent epithelial loss, capillary rarefaction, and progressive fibrosis due to ECM deposition. Efferocytosis is a central mechanism in orchestrating the balance between inflammation and repair after renal tissue injury. Both TECs and macrophages play a part in this dynamic and staged process. TECs are the predominant cell type during the early injury phase, while macrophages are essential in the later stages. Effective coordination between these cell types helps maintain tissue homeostasis, whereas impairment in efferocytosis leads to prolonged inflammation, maladaptive repair, and fibrosis 176.

Upon injury, an early biomarker of AKI, kidney injury molecule-1 (KIM-1), is upregulated in dedifferentiated TECs. KIM-1 is a phosphatidylserine receptor that enables recognition of apoptotic cells and oxidized lipids and promotes their engulfment. The epithelial clearance mechanism initiates local resolution by reducing the release of DAMPs and dampening pro-inflammatory signaling. KIM-1 activation triggers phosphorylation events to recruit the PI3K p85 subunit and activate the PI3K/AKT cascade, which in turn inhibits IκB kinase (IKK)/NF-κB signaling and reduces the production of TNF-α, IL-6, and MCP-1. Concurrently, ERK/MAPK signaling promotes epithelial migration and proliferation, facilitating tissue repair 177,178. In addition, properdin, secreted by TECs, supports efferocytosis by promoting autonomous uptake of apoptotic debris by TECs, reducing the release of DAMPs like High Mobility Group Box 1, and attenuating inflammatory responses. In the properdin-deficient mice, efferocytosis by TECs is impaired, leading to enhanced apoptosis and impaired renal function in an IRI model 179.

As the injury progresses and the initial epithelial response wanes, macrophages become the principal phagocytic population. Macrophages transition into a reparative (M2-like) phenotype, and they clear apoptotic cells, degrade excess ECM via MMPs, and secrete anti-inflammatory cytokines such as IL10. These activities help resolve inflammation and promote TECs proliferation, angiogenesis and tissue regeneration, thereby preventing irreversible glomerular damage and facilitating renal repair 180. TREM2, an upstream immune-regulatory receptor, plays a crucial role in efferocytosis. By activating the PI3K/AKT pathway, TREM2 enhances the clearance of apoptotic cells by macrophages and suppresses the production of pro-inflammatory cytokines (e.g., TNF-α, IL-6), ultimately mitigating the progression from AKI to chronic kidney diseases. Overexpressed TREM2 on macrophages can improve survival and promote repair, while a deficiency in TREM2 exacerbates inflammation, leading to increased fibrosis and tubular atrophy 181. Additionally, V-domain Ig suppressor of T cell activation (VISTA), an immune-checkpoint molecule that is predominantly expressed on macrophages, simultaneously enhances macrophage efferocytosis of apoptotic cells and suppresses excessive T-cell activation. During the repair phase after renal ischemic injury, VISTA-positive resident macrophages accelerate tissue recovery through this dual mechanism of debris clearance and immunosuppression 182.

Kidney fibrosis results from maladaptive repair due to repeated or severe injury in which the normally self-limiting wound-healing program becomes chronic. In addition to repeated or severe injury, kidney fibrosis stems from disruption in the phagocytic crosstalk, and the failure in efferocytosis converts a self-healing process into a fibrosis process to form a matrix-laden scar. A few molecules have been identified as inhibitors of effective efferocytosis, leading to exacerbated kidney injury and progression to fibrosis. For instance, junctional adhesion molecule-like protein (JAML), which is upregulated on macrophages during AKI, promotes the conversion of macrophages into a pro-inflammatory M1 phenotype with a reduced efferocytosis capacity. Therefore, upregulation of JAML prolongs tissue damage by preventing efficient clearance of apoptotic cells and perpetuating inflammation. In the *JAML^-/-^* mice, JAML deficiency attenuates inflammatory responses by impeding the infiltration of macrophages and neutrophils and reducing the level of proinflammatory mediators in the kidney from the mice with renal IRI 183. Additionally, microRNAs, such as miR-126, have been shown to impair efferocytosis by inhibiting the PI3K/insulin receptor substrate 1 (IRS-1)/focal adhesion kinase (FAK) signaling pathway and promoting M1 polarization. Impaired efferocytosis accelerates the fibrotic process by inhibiting the anti-inflammatory and reparative roles of macrophages 184. The macrophage receptor CD36 can facilitate apoptotic cell clearance, paradoxically, CD36 also promotes fibrosis by activating oxidative fibrogenic signaling pathways during chronic kidney injury. CD36 triggers NADPH-oxidase-dependent ROS production and subsequent activation of the TGF-β/Smad and NF-κB pathways. The oxidative burst skews macrophages toward a profibrotic M2-like phenotype, stimulates myofibroblast differentiation, and accelerates ECM accumulation during chronic kidney injury 185.

All lupus nephritis (LN) patients demonstrated overwhelming tubulointerstitial injury in the acute inflammatory period, with clear tubular necrosis and acute inflammatory cell infiltration. In this sense, the acute phase of LN could also be viewed as a type of AKI. Defective efferocytosis participates in the immunopathogenesis of LN. Impaired efferocytosis of macrophages result in the persistent accumulation of apoptotic bodies in the renal tissue and the formation/deposition of immune complexes in glomeruli. The accumulated immune complexes cause complement activation and amplify the local inflammation and induce extra renal injury. Molecular basis of defective clearance includes downregulated expression of scavenger receptors and impaired regulation of PPAR-γ/retinoid X receptor α (RXRα) signaling pathways in macrophages. PPAR-γ/RXRα signaling pathways are a transcriptional program that normally drives the engulfment machinery 186. In LN, metabolic support for efferocytosis is also compromised. Carnitine palmitoyl transferase (CPT1a), a key enzyme located on the outer mitochondrial membrane, is essential for the metabolic state and function of macrophages, such as their polarization profile and efferocytotic activity. When the CPT1a level in macrophages is sufficiently low, macrophages do not have adequate fatty-acid-oxidation (FAO)-derived energy, their efferocytosis capacity is reduced, and apoptotic debris accumulates, thereby intensifying inflammation in LN 187. Additionally, enhanced activation of the tonicity-responsive enhancer-binding protein (TonEBP), a transcription factor highly responsive to osmotic stress and inflammatory signals, exacerbates inflammation in LN by inhibiting efferocytosis and simultaneously enhancing antigen presentation, perpetuating autoimmune responses 188. Conversely, enhancing macrophage-mediated clearance pathways, such as LC3-associated efferocytosis, can attenuate autoimmunity and improve outcomes. Furthermore, therapeutic modulation of the macrophage function through exosomal microRNAs, such as miR-16 and miR-21 derived from mesenchymal stem cells (MSCs), has shown promising outcomes by shifting macrophages towards an anti-inflammatory, pro-efferocytotic phenotype. These macrophages efficiently clear apoptotic debris and promote regulatory T-cell recruitment (**Figure 4**C) 189.

### 3.6 Efferocytosis in skeletal muscle injury

Skeletal muscle is one of the few adult mammalian tissues that can restore its structure and rehabilitate the contractile performance after substantial injury. Muscle regeneration is characterized by a well-coordinated sequence of events that involves immune cell infiltration, necrotic fiber clearance, activation of satellite cells (SCs), and remodeling of the extracellular matrix. At the core of this regenerative process are SCs, the paired box protein 7** (**PAX7)-positive muscle stem cells, which reside beneath the basal lamina of each myofiber. In a healthy muscle, they are mitotically quiescent sentinels, but within hours of injury they receive biochemical and mechanical cues and they are driven through a tightly choreographed cascade of events: activation, clonal expansion, lineage commitment, terminal differentiation into myoblasts, and ultimately fusion either with each other or with existing myofibers. This cascade is essential for re-establishing intact, multinucleated fibers capable of generating force. It is noted that during this cascade of events, SCs are governed by a transient but highly organized inflammatory milieu dominated by macrophages, while the function of macrophages is controlled by efferocytosis 190. The interweaving relationship between efferocytosis and SC biology is pivotal to manipulating skeletal-muscle repair.

The regenerative process is conventionally divided into three overlapping phases—degeneration/inflammation, regeneration, and remodeling. A laceration, toxin injection, or an ischemic insult causes local myofiber necrosis and vascular leakage, thereby releasing DAMPs to trigger complement activation and endothelial adhesion-molecule expression. Neutrophils swiftly arrive at the injured site. They execute valuable debris clearance and antimicrobial tasks, while their persistence is harmful for muscle regeneration. Timely removal of apoptotic neutrophils is crucial. Roughly 12-24 h post-insult, Ly6C^hi^/CCR2^+^ monocytes respond to the CCL2 gradient generated from both injured fibres and resident immune cells. Lu et al. have shown that CCL2 expression is essential for both monocytes and the muscle, and CCL2 gradient triggers effective recruitment of monocytes into the injured site, and subsequent secretion of macrophage-derived insulin-like growth factor 1 (IGF-1), a trophic factor that directly stimulates SC expansion. The M1-like macrophages, the first wave of recruited cells, secrete TNF-α, IL-1β, IL-6, nitric oxide (NO), and MCP-1, collectively activating quiescent SCs, up-regulating the myogenic differentiation 1/myogenic factor 5 (MyoD/MyF5), and driving SCs proliferation 191.

To engulf apoptotic neutrophils and necrotic fibre fragments, macrophages sense external PS via multiple receptor systems. Recognition of “eat-me” signals (e.g., external PS) on dying cells re-wires the signaling network in macrophages, reprogramming their transcriptome toward an anti-inflammatory, M2-like phenotype. This transition plays a vital role in productive muscle healing since persistent inflammation, fibrotic deposition, and functional decline are often associated with the presence of the M1-like macrophages. An array of receptor-ligand pairs mediate PS sensing, while the TAM family tyrosine kinases, including Tyro3, Axl, and especially MerTK, play a dominant role in skeletal muscle. Up-regulation of Mer coincides with the phenotype M1-to-M2 conversion; a deficiency in MerTK hinders apoptotic-cell clearance and prolongs the cytokine storm, ultimately stalling SC-driven myogenesis 192. Intracellularly, AMPKα1, an energy sensor protein regulating cellular energy metabolism, play a role in phagocytic signaling. The deletion of AMPKα1 in myeloid cells inhibits efferocytosis-induced M2 polarization and markedly delays the regeneration process 193. In addition to these kinase pathways, the Ras homolog family member A-Rho-associated coiled-coil-containing protein kinase 1 (RhoA-ROCK1) signaling axis controls the formation of the phagocytic cup by regulating cytoskeletal dynamics. Inhibition of this pathway paradoxically enhances efferocytosis and induces the production of the nuclear factor I X (NFIX), a transcription factor, which in turn reinforces the M2 transcriptional signature. The deletion of NFIX in myeloid cells locks macrophages into an inflammatory phenotype and induces defective SC differentiation 194.

Upon the completion of efferocytosis, macrophages are transformed into a trophic factory. Their secretome orchestrates the subsequent SC fate. Growth differentiation factor-3 (GDF3) and IGF-1 act in synergy to accelerate myoblast alignment and fusion, thus promoting the expansion of regenerating fibers. TGF-β and IL-10 resolve residual inflammation, and they mitigate excessive proliferation of SCs, steering the SC progeny toward quiescence to replenish the stem-cell pool 195. Specific pro-resolving lipid mediators, such as Resolvin D1 (RvD1), bind to G-protein-coupled receptors to boost efferocytosis and transform macrophages toward a CD206^+^/Ly6C^lo^ phenotype. Markworth et al. have demonstrated that systemic RvD1 not only accelerates neutrophil clearance but also helps the growth of regenerated fibers and the generation of the contractile force. The performance of these lipid mediators is heavily dependent on opsonins like MFG-E8, which provide a bridge between PS on apoptotic bodies and integrins on phagocytes. The retinol saturase (RetSat)-MFG-E8 axis is a great example of the opsonins. Macrophages without RetSat barely produce MFG-E8 and engulf fewer apoptotic bodies, consequently, the M2 polarization is not activated. SCs may be able to sense the deficiency in the cytokine and initiate compensatory actions, but the niche remains sub-optimal, yielding smaller, mis-aligned fibers and patchy ECM 196. Additionally, transglutaminase 2 (TG2) acts as a PS co-receptor to facilitate apoptotic-cell tethering and an intracellular cross-linker to promote myoblast fusion. Inside differentiating myogenic cells, TG2 establishes cross-links between ECM proteins and fusogenic factors. Therefore, in TG2-null mice, the majority of apoptotic bodies are initially cleared, while the release of the downstream growth differentiation factor 3 (GDF3) and the myotube fusion are compromised, yielding thinner, weaker fibers, which indicates that TG2 has a dual role in macrophage signaling and myogenic cell morphogenesis 197.

Between 2 days and 5 days post injury, the SC progeny accumulates as proliferating myoblasts. At the injury site, M2 macrophages and their cytokines instruct the SC progeny to switch from proliferation to differentiation 198. By day 7, while efferocytosis is maintained to prevent residual fibrin or apoptotic debris from physically obstructing fusion, macrophages simultaneously secrete the matrix MMP and pro-angiogenic VEGF to recruit endothelial cells and fibro/adipogenic progenitors to remodel the ECM. From day 10 onwards, as myotubes mature and switch to an adult MyHC isoform, the macrophage population declines, but efficient late-phase efferocytosis remains to be vital. Uncleared apoptotic bodies can calcify or incite fibrosis via chronic TGF-β release. Instead, successful clearance of apoptotic bodies promotes vascular and neural ingrowth and restores the mechanical continuum between tendon and regenerated muscle 198.

Under an aged or metabolically diseased condition, the AMPK activity declines, and the MerTK signaling is weakened, leading to inefficient phagocytosis of CX3CR1^+^ macrophages. Runyan et al. have shown that influenza-infected injury in the old mice impairs the regeneration process because resident macrophages cannot downregulate the MHC-II expression and SCs cannot proliferate 199,200. Similar observations are found in the MerTK-null mice or when CCR2 or CCL2 is pharmacologically blocked, suggesting risks may be associated with chronic anti-chemokine therapies** (Figure 4D).**

## 4. Strategies for promoting tissue regeneration via targeting efferocytosis

**Figure 5:**
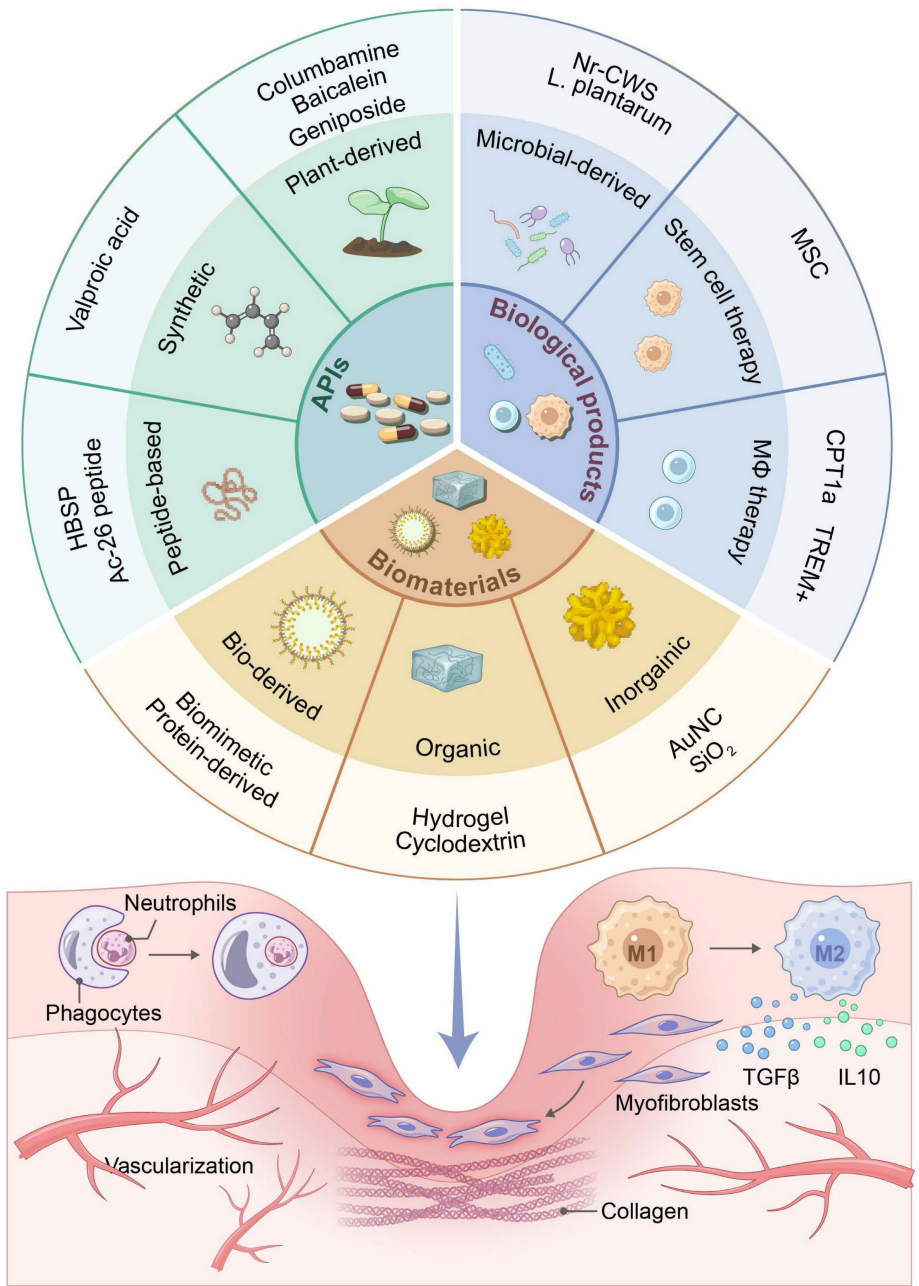
** Current therapeutic strategies for tissue regeneration via targeting efferocytosis**. These strategies are categorized into three major modalities: (1) small molecular APIs including plant-derived compounds (e.g., columbamine, baicalein, geniposide), synthetic drugs (e.g., valproic acid), and peptides (e.g., Ac-26 peptide, HBSP); (2) Biologicals, including microbial agents (Nr-CWS, *L. plantarum*), stem cells, and MΦs (e.g., CPT1α⁺ MΦs, TREM⁺ MΦs); and (3) Biomaterials, including bio-derived materials (e.g., biomimetic vesicles, protein-based materials), organic materials (e.g., hydrogels, cyclodextrins), and inorganic materials (e.g., AuNCs, SiO₂ nanoparticles). Cellular interactions between neutrophils, phagocytes, M1/M2 macrophages, and myofibroblasts, and key processes including vascularization, TGFβ/IL10 signaling, and collagen deposition are schematically described in the lower section. These therapeutic modalities modulate tissue repair: phagocytosis of necrotic neutrophils by macrophages; polarization of M1 (pro-inflammatory) macrophages to their M2 phenotype (anti-inflammatory); activation of myofibroblasts to secrete collagen regulated by TGFβ and IL10; and mediation of vascularization by endothelial cells. APIs: Active pharmaceutical ingredients; HBSP: helix B surface peptide helix B surface peptide; Nr-CWS: nocardia rubra cell wall skeleton; L. plantarum: Lactiplantibacillus plantarum; MSC: mesenchymal stem cell; MΦ: Macrophage; AuNC: Gold nanocage.

Given the central role of efferocytosis in inflammation resolution and tissue repair, innovative and promising regenerative therapeutic strategies for promoting ACs clearance have emerged. Diverse modalities have been developed to modulate efferocytosis pathways, including small synthetic molecules, peptides, plant-derived compounds, microbe-derived agents, cell and cell-derived therapeutics, and engineered biomaterial systems (**Figure 5** and** Table 2**). While preclinical studies have demonstrated significant potential of these modalities for tissue regeneration, there are very few clinical trials on tissue regeneration because of a few challenges described below. Current trials are predominantly focused on oncology and metabolic disorders 201-204.

The challenges for clinical trials on tissue regeneration stem from three interconnected barriers: mechanistic complexity, technical limitations, and clinical translation challenges. 1) Mechanistic complexity in regeneration. Tissue repair requires precise spatiotemporal coordination between efferocytosis-mediated inflammation resolution and regenerative processes (e.g., stem cell differentiation, ECM remodeling). Different from cancer/metabolic diseases with distinct “on/off” targets (e.g., CD47 blockade), balanced modulation across multiple phases is essential for tissue regeneration. An excessive suppression level of inflammation may impair pro-regenerative signals. For instance, IL10 overexpression can delay neutrophil recruitment, compromising early repair. Paradoxically, efferocytosis may drive fibrosis in a specific context. Enhanced efferocytosis can activate profibrotic pathways (e.g., TGF-β release from macrophages), inadvertently accelerating the formation of pathological scars. 2) Technical barriers in delivery and assessment. Current carriers (e.g., liposomes, hydrogels) cannot precisely, sequentially release therapeutics in the corresponding phases including inflammation, proliferation and remodeling. Smart scaffolds (e.g., ROS/pH-responsive hydrogels) have been developed while they are in the preclinical stage. 3) Clinical translation hurdles. While animal models for cancer and metabolic disorders established from humanized technologies have demonstrated their translational potential, large-organ regeneration models face inherent complexity in accurately simulating human microenvironments (e.g., vascularization, neural integration) and the risk of overestimating efficacy in small animals. For instance, zebrafish and murine models exhibit potent cardiac regeneration post-injury, whereas human infarcts are found to have a negligible regenerative capacity 205. Furthermore, the commercial viability of tissue regeneration therapies lags behind that of cancer and metabolic drugs. The cancer/metabolic drugs possess a competitive advantage in terms of pricing, and they are often supported by robust reimbursement systems. In addition, they have shorter timelines for efficacy validation, typically requiring 1-3 years of clinical data, however, 5-10 years as regenerative endpoints are required for tissue regeneration therapies. Nevertheless, targeting efferocytosis for tissue repair has shown its therapeutic promise. The mechanistic and translational studies on efferocytosis for tissue repair have offered novel approaches to addressing the above hurdles, eventually paving the way for harnessing clearance pathways for tissue regeneration. In the following section, we systematically survey reported therapeutic approaches, categorized by their modality, to provide a comprehensive analysis of current progress and prospectives.

### 4.1 Active pharmaceutical ingredients

Active pharmaceutical ingredients (APIs) are the biologically active components of medicinal products responsible for their therapeutic effects. They exhibit three core characteristics: 1) Targeted bioactivity through direct interaction with specific molecular pathways (e.g., efferocytosis receptors, metabolic enzymes) to modulate disease procession; 2) Chemical precision with a high purity and a defined structure-activity relationship to predict dose-response outcomes; 3) Therapeutic advantages of precise controllability in modulating complex biological cascades to facilitate tailored regenerative interventions 206. To date, APIs reported to enhance efferocytosis for tissue repair include synthetic small molecules, peptides, and plant-derived compounds.

Synthetic small molecule pharmaceuticals are often employed as precision-engineered molecular modulators to target specific efferocytosis pathways. Their high target specificity and dose-response predictability have established a controllable foundation for regenerative modulation 207. Valproic acid (VPA), a branched short-chain fatty acid, is an inhibitor for histone deacetylase (HDAC). VPA enhances efferocytosis by upregulating phagocytosis-associated molecules (e.g., CD36, MerTK, MFGE8 and Gas-6) while simultaneously inhibiting the PI3K/AKT/murine double minute 2 (MDM2) signaling pathway in macrophages. This dual action suppresses NF-κB-mediated inflammatory activation and promotes macrophage-mediated clearance of apoptotic cells, thereby accelerating collagen deposition, granulation tissue development, wound healing, and epithelialization 208. In addition to VPA, topical insulin and aspirin trigger metabolic reprogramming for targeting efferocytosis. Insulin targets phagocytosis-induced apoptosis, while aspirin modulates arachidonic acid metabolism toward pro-resolving lipids. These agents enhance macrophage efferocytosis by upregulating the expression of CD36 receptors, reducing the accumulation of neutrophils, and promoting the polarization of macrophages toward an anti-inflammatory M2 phenotype from an M1 phenotype. Eventually, these agents help achieve inflammation resolution, collagen deposition, and wound closure in diabetic wounds 209,210.

Helix B surface peptide (HBSP) and ANXA1-derived peptide Ac2-26 are two representative examples of peptides for targeting efferocytosis. HBSP, a 15-amino acid derivative of the erythropoietin structural domain, has been explored to target the innate repair receptor, the EPOR/βcR complex, on renal tubular epithelial cells (TECs) to enhance phagocytic clearance of apoptotic debris and improve renal repair after IRI in the properdin-deficient mice 211. Ac2-26, a biologically active N-terminal fragment of annexin A1 (AnxA1), can mimic the anti-inflammatory action of AnxA1 by activating formyl peptide receptors, particularly formyl peptide receptor 2 (FPR2). Ac2-26 functions as a PS-bridging molecule to facilitate PS recognition by macrophage FPR2 receptors in the efferocytosis recognition phase, attenuate neutrophil infiltration, and promote macrophage recruitment and polarization toward an immunoregulatory M2 phenotype to collectively accelerate diabetic wound healing 212.

Plant-derived compounds, originating from natural sources, offer a multi-target approach to modulating efferocytosis by simultaneously acting on interconnected pathways, thereby providing synergistic effects to address the complexity of impaired tissue repair under a chronic condition 213. Among these compounds, isoliquiritigenin (ISL), a flavonoid from *Glycyrrhiza* species (licorice), enhances dendritic cell efferocytosis through SLC7A11-glycolysis regulation, improving the endothelial function and accelerating diabetic wound healing 214. In addition, baicalein, a *Scutellaria baicalensis* flavonoid, upregulates the expression of macrophage TREM2 via the tropomyosin receptor kinase B-cAMP response element-binding protein 1 (TrKB-CREB1) pathway to drive M2 polarization and promote efferocytosis, suppressing acute inflammatory injury in liver failure 215. Columbamine (COL), a natural alkaloid, acts as an efferocytosis enhancer by targeting formyl peptide receptor 2 (FPR2), a GPCR. COL can promote LC3-associated phagocytosis (LAP) by recruiting LC3 to phagosomes and accelerating degradation of ACs, thereby alleviating intestinal inflammation 216. Geniposide, an iridoid glycoside from *Gardenia jasminoides*, ameliorates ventilator-induced acute kidney injury by enhancing macrophage efferocytosis of neutrophil extracellular traps (NETs) through activation of AMPK-PI3K/AKT and upregulation of efferocytosis receptors CD31/CD44 217. Ginsenoside Rg5, a natural compound from *Panax ginseng*, directly binds to and inhibits SLC7A11, blocking cystine uptake to enhance glycogenolysis and glycolysis in dendritic cells, which fuels efferocytosis, boosts apoptotic cell clearance, reduces inflammation, and accelerates wound repair 218. Finally, Naoxintong, a traditional Chinese medicinal formulation composed of 16 herbal, resin, and animal-derived ingredients, enhances macrophage efferocytosis of neutrophils, promotes M2 polarization, and alleviates diabetic wound inflammation via STAT3/STAT6 activation 219.

### 4.2 Biologicals

Biologicals (e.g., proteins, nucleic acids, whole cells) are therapeutic agents produced from living or freshly killed organisms or parts of organisms. They display a highly complex structure and they are produced using biotechnological processes, in contrast to chemically synthesised drugs 220. The biologicals achieve high target specificity due to their molecular recognition (e.g. antibody-antigen specific binding) and can effectively modulate the proximal disease pathway while limiting off-target toxicity compared with synthetic drugs. Moreover, the biologicals' multi-pathway modulation potential is realized when they engage in overlapping biological cascades. Because of their high molecular weight (>5 kDa), the biologicals avoid being rapidly cleared through the kidneys and can maintain their therapeutic effect for weeks, which is dramatically longer than small-molecule drugs 221. Unlike synthetically engineered biomaterials, biologicals primarily function as “instructive signals” or “effector units**”** that directly modulate cellular processes like efferocytosis through biological recognition and signaling 222. To date, the use of biologicals derived from microorganisms and mammalian cells to enhance efferocytosis for wound healing have been reported.

Microbial-derived agents leverage the immunomodulatory properties of microorganisms or their products to enhance efferocytosis. By interacting with the host immune system and the microbiota in injured sites, these microbial-derived agents can create a conducive microenvironment for tissue regeneration and repair 223. The nocardia rubra cell wall skeleton (Nr-CWS), an immunomodulator extracted from the gram-positive bacterium *Nocardia rubra*, enhances wound healing through coordinated immunomodulation and cellular reprogramming. By recruiting and activating macrophages at the wound site, Nr-CWS significantly boosts macrophage proliferation, migration, and efferocytosis. Crucially, it polarizes macrophages toward a pro-healing M2 phenotype via the activation of the PI3K/Akt/mTOR pathway. *In vivo*, Nr-CWS-treated wounds exhibit accelerated deposition of collagen, an elevated level of M2 macrophage infiltration and improved wound closure 224. Meanwhile, the supernatant from* Lactiplantibacillus plantarum* (*L. plantarum*) enhances diabetic wound repair by boosting polymorphonuclear phagocytosis in patients, reprogramming the macrophage phenotype, stimulating angiogenesis and collagen deposition, and reducing bacterial accumulation, collectively improving foot ulcer debridement outcomes 225.

Cell therapies using stem cells and macrophages represent a promising frontier in regenerative medicine. MSCs are stimulated to secrete MFG-E8 to reduce the production of TNF-α⁺ and help phagocytosis of apoptotic cells in diabetic wounds 226. Intraperitoneal hMSCs are used to resolve inflammation and promote tissue repair via PGE2-mediated T-cell suppression and efferocytosis-driven macrophage reprogramming toward an M2 phenotype in the Crohn's disease 227. In carbon tetrachloride-induced liver injury, MSCs have been explored to restore macrophage homeostasis by converting Ly6C pro-inflammatory monocytes into phagocytotically competent Ly6C monocyte-derived macrophages (MoMF I) with upregulated lysosomal genes including cathepsin B (CTSB), cathepsin D (CTSD), and lysosomal-associated membrane protein 1 (LAMP1) 228.

The macrophage-directed therapy is realized through monocyte reprogramming using the human amniotic mesenchymal cell-conditioned medium (hAMTC-CM). M2-like macrophages are generated with elevated levels of phagocytosis and IL10 production, accelerating wound regeneration 229. The alternatively activated macrophages (AAMs) promote the resolution of acetaminophen-induced acute liver injury by directly clearing necrotic debris, suppressing inflammation, and stimulating regeneration 230. Genetic engineering is often employed to overexpress specific genes in macrophages to enhance phagocytosis. For example, CPT1a, a key enzyme in fatty acid oxidation, is overexpressed in macrophages, and they are reprogramed to improve phagocytosis in fibrotic kidneys and significantly reduce renal fibrosis in unilateral ureteral obstruction (UUO) mice 187; Macrophages with upregulated TREM2⁺ via HIF-1α during AKI-CKD progression can improve efferocytosis upon adoptive transfer, mitigating tubular damage in urine IRI models 181; Bone marrow-derived macrophages (BMDMs) engineered with adenoviral heme oxygenase-1 (HO-1) accelerate clearance of renal microvascular platelet aggregates in the renal medullary microvasculature, improving the renal function in IRI 231.

### 4.3 Biomaterials-assisted therapies

Biomaterials have been developed for efferocytosis-targeting therapies, and advances in nanotechnology and material science have been harnessed to overcome limitations associated with targeting efferocytosis. They are natural or synthetic materials engineered to interact with biological systems. Their primary role in efferocytosis modulation is to provide structural support, serve as controlled-release depots for therapeutic agents (including biologicals), and engineer the physicochemical microenvironment to favor reparative processes. They can be conceptualized as the “scaffolding and logistics infrastructure” that supports and enhances the function of biologicals and endogenous cells 232-239.

To systematically elaborate breakthroughs in the biomaterials-assisted therapies, we categorize the biomaterials systems into organic, inorganic, bio-derived, biomimetic, and multicomponent hybrids based on their chemical composition. Each category exhibit distinct physicochemical properties that dictate their interactions with biological systems and therapeutic efficacy.

#### 4.3.1 Organic biomaterials

Organic biomaterials are biocompatible substances derived from natural or synthetic organic polymers and they have been used to construct foundational platforms in regenerative medicine due to their biomimetic properties, tunable biodegradability, and dynamic responsiveness to physiological cues (e.g., pH, temperature) 240.

Hydrogels, a prominent organic biomaterial type, are formed through crosslinking of hydrophilic polymer networks via covalent bonds or physical interactions (e.g., hydrogen bonds). Drugs can be encapsulated inside the interconnected pores of a hydrogel and drug release is achieved when the hydrogel is swollen in an aqueous environment. Enhanced molecular diffusion within the hydrogel enables tailored drug delivery (e.g., growth factors, antibiotics) to the wound site. Meanwhile, the mechanical strength of a hydrogel can be tuned for a specific tissue for cell support. Therefore, hydrogels have been widely explored for wound healing and tissue engineering 241-243. For example, the modified collagen gel (MCG), a bovine collagen-derived hydrogel dressing, displays targeted immunomodulation by promoting macrophage efferocytosis through the activation of the miR-21-JNK-IL10 signaling axis, thereby stimulating vascularization in ischemic wounds 70.

**Figure 6:**
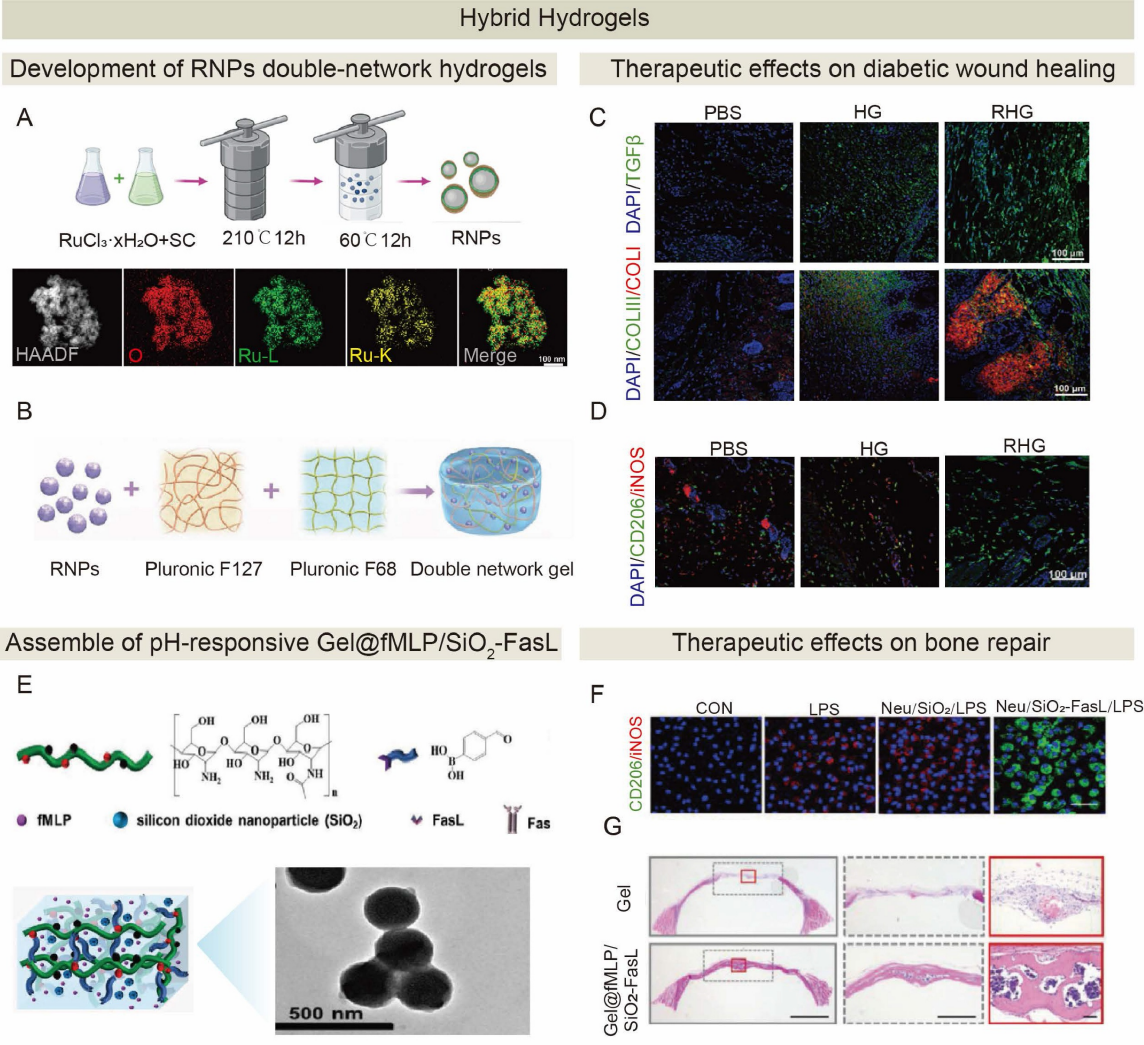
**Hybrid hydrogels for immunomodulation and tissue regeneration.** (A) Synthesis and characterization of RNPs. Adapted from ref.^246^. (B) Assembly of a double-network thermosensitive hydrogel loaded with RNPs. Adapted from ref.^246^. (C) Fluorescence images of wound sections stained for collagen I (COLI, red), collagen III (COLIII, green), and nuclei (DAPI, blue). RGH treatment significantly enhanced collagen deposition and organization compared to controls. Adapted from ref.^246^ (D) Immunofluorescence staining of macrophage markers: anti-inflammatory M2 phenotype (CD206, green) and pro-inflammatory M1 phenotype (iNOS, red). RGH promoted M2 macrophage polarization. Adapted from ref.^246^ Licensed under Creative Commons CC-BY-NC-ND license. (E) Design of a pH-responsive hybrid hydrogel, Gel@fMLP/SiO₂-FasL, for neutrophil recruitment and clearance. Adapted from ref.^247^. (F) Representative fluorescence images of macrophage phenotypes after incubation with different pretreated neutrophils; iNOS (red), CD206 (green), and nuclei (blue). Adapted from ref.^247^. (G) H&E-stained images to support new bone formation and matrix deposition after treatment with Gel@fMLP/SiO₂-FasL. Adapted from ref.^247^. Licensed under Creative Commons CC BY license. RNPs: RuO₂ nanoparticles; HG: Pluronic F127 and F68 hydrogel; RHG: RNPs-Hydrogel; SC: sodium citrate; HAADF: High-angle annular dark-field; fMLP: Formyl-met-leu-phe; SiO₂-FasL: FasL-conjugated silica dioxide nanoparticles; Gel@fMLP/SiO₂-FasL: a phenylboronic acid -based polymeric hydrogel loaded with fMLP/SiO_2_-FasL complexes.

To enhance the hydrogel functionality, hybrid hydrogels have been developed by integrating polymeric matrices (e.g., gelatin, collagen, fibrin) with nanomaterials (e.g., nanoparticles, exosomes) to overcome limitations of single component hydrogels. By leveraging synergistic interactions between organic matrices and inorganic/organic nanofillers, these hybrid hydrogel platforms create a dynamic, bioactive scaffold for tissue repair. Specifically, they can mimic a 3D extracellular matrix (ECM) environment, thereby promoting cellular adhesion, proliferation, and survival of encapsulated cells while enabling controlled therapeutic drug delivery 244. For instance, a pH-responsive polyvinyl alcohol (PVA)/gelatin hydrogel was loaded with lipid nanoparticles modified with tiliroside (Til)-conjugated iRGD&PS to construct iRGD&PS@PLGA NPs. After the hydrogel was exposed to an acidic wound microenvironments (pH 5.0-6.0), it underwent rapid degradation to realize on-demand nanoparticle release. Dual-targeting strategies were employed in the hydrogel. The iRGD peptide bound to αvβ3/αvβ5 integrins on the inflamed endothelium, and PS targeted MerTK/TIM-4 receptors on macrophages to enhance cellular uptake. Upon internalization, Til inhibited both SLC7A11 and pyruvate kinase isozyme M2 (PKM2), thereby disrupting the cystine-glutamate exchange and the glycolytic flux. Consequently, this dual action boosted efferocytosis by enhancing apoptotic neutrophil clearance and reprogramming macrophages toward a glycolysis-dependent repair phenotype (M2 polarization), ultimately suppressing inflammation and promoting angiogenesis. In a diabetic murine model, this hybrid hydrogel accelerated wound healing by resolving chronic inflammation and restoring metabolic homeostasis 245. In addition, Ruthenium (IV) oxide nanoparticles (RNPs), synthesized via hydrothermal treatment, were embedded in a Pluronic F127/F68 double-network hydrogel. The hybrid hydrogel exhibited three unique features: thermosensitivity with rapid sol-gel transition at 31°C for precise interfacial conformation at the wound site; injectable self-healing through shear-thinning of the hydrogel to achieve deep wound penetration; and biological activity of triggering TGF-β1 signaling in fibroblasts by RNPs. Fibroblasts were activated for ECM reconstruction and stimulated for secretion of macrophage colony-stimulating factor (M-CSF), which upregulated the expression of the macrophage efferocytosis receptors (MerTK/TIM4) to accelerate diabetic wound healing 246. Another pH-responsive hybrid hydrogel, Gel@fMLP/SiO₂-FasL, was prepared to accelerate refractory wound healing by inducing a transiently heightened inflammatory response. Spatiotemporal control release of drugs from this hydrogel was realized. Burst release of fMLP, a neutrophil chemoattractant, initiated acute inflammation, and acid-triggered degradation of the hydrogel structure facilitated the release of SiO₂-FasL nanoparticles to induce neutrophil apoptosis via Fas signaling. This cascade release strategy promoted macrophage efferocytosis and anti-inflammatory polarization, resolving inflammation while driving regeneration. Notably, in diabetic wounds, the hybrid hydrogel accelerated wound closure. Interestingly, in critical-sized bone defects, the hydrogel helped regenerate the bone, demonstrating broad therapeutic potential of the hybrid hydrogel (**Figure 6**) 247.

**Figure 7:**
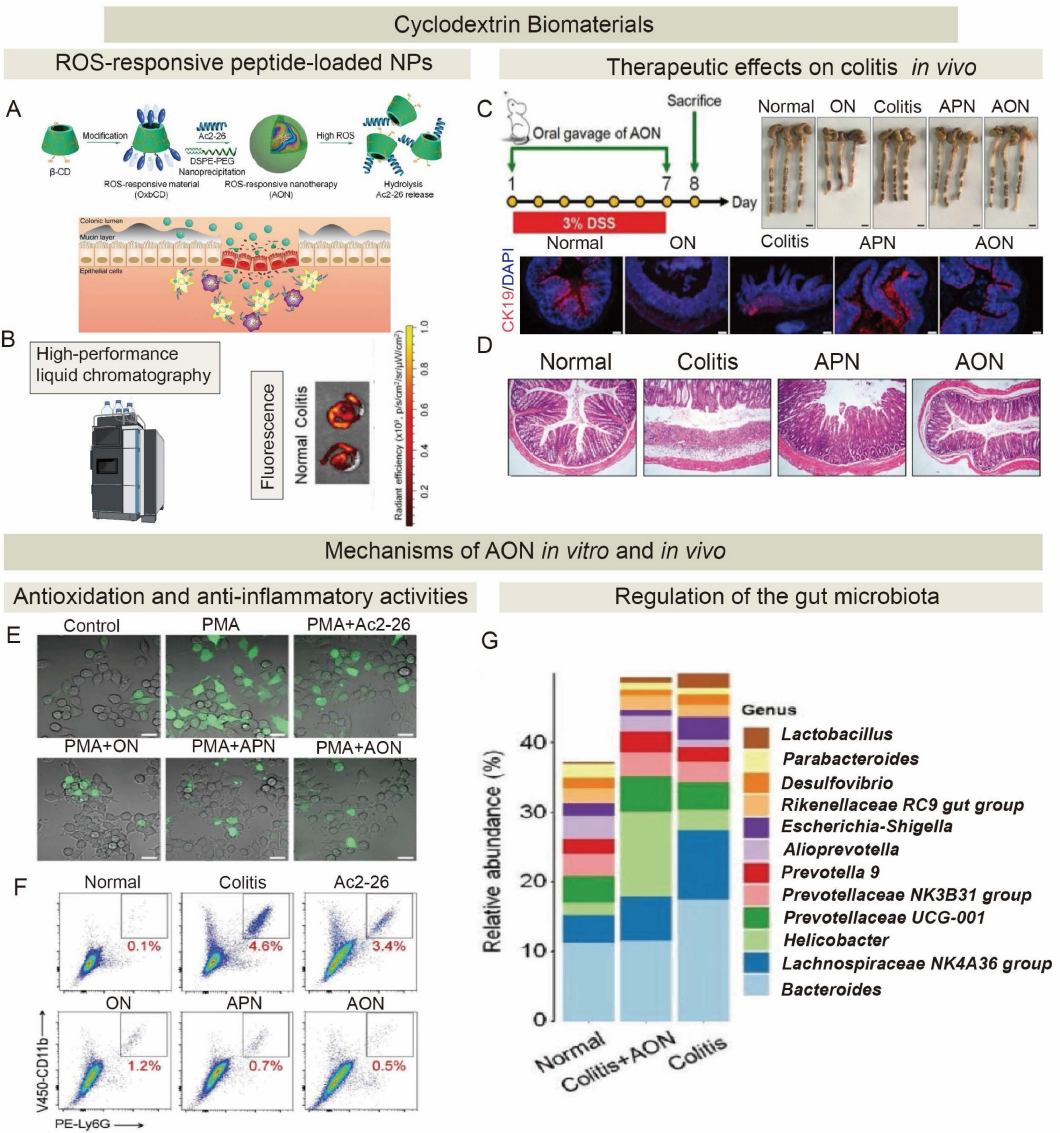
** Development and therapeutic evaluation of ROS-responsive peptide-loaded nanoparticles for targeted treatment of colitis.** (A) Schematic design and synthesis of ROS-responsive nanoparticles, AON. (B) *In vivo* biodistribution and colon-targeting capability of AON. Fluorescence images supported significantly enhanced accumulation of AON in colitis tissues compared to the normal colon at 24 h post-administration. (C) Macroscopic evaluation of therapeutic efficacy of AON in acute colitis and representative immunofluorescence images of colon sections stained for CK19 (red) and DAPI (blue). (D) Histopathological assessment of chronic colitis after AON treatment. (E) Antioxidant effects of AON *in vitro*. (F) Neutrophil infiltration analysis by flow cytometry. AON treatment significantly reduced neutrophil infiltration in colitis mice (**P<0.01). (G) Gut microbiota modulation. AON treatment restored the *Prevotellaceae* population in colitis mice. Data are presented as mean ± SEM. Statistical significance was determined by one-way ANOVA with Tukey's post-hoc test. Adapted from ref.^249^. Licensed under Creative Commons CC-BY license. β-CD: β-cyclodextrin; DSPE-PEG: distearoylphosphatidylethanolamine-polyethylene glycol; DSS: dextran sulfate sodium; ON: empty nanoparticles; APN: non-ROS-responsive Ac2-26 nanoparticles; AON: oxidation-responsive nanoparticles containing a proresolving annexin A1-mimetic peptide Ac2-26.

Cyclodextrins (CDs) are cyclic oligosaccharides (α-, β-, or γ-CD) composed of glucose units. A hydrophobic cavity and a hydrophilic exterior are formed in CDs. This unique structure endows them to function as a molecular carrier by encapsulating therapeutic agents via host-guest interactions or a structural scaffold for cell support. CDs have advantages including superior drug solubilization, stimuli-responsive release (e.g., pH/ROS-triggered payload delivery), low immunogenicity, and minimized systemic toxicity through renal clearance. CDs have been explored for targeted regenerative therapies because of their biocompatibility and functionalization versatility (e.g., grafting onto polymers or nanoparticles) 248. For example, an oral nanotherapeutic formulation, AON, was derived from ROS-responsive oxidation-labile β-cyclodextrin (OxbCD). The nanoparticles were engineered from OxbCD after encapsulation of the anti-inflammatory peptide Ac2-26. The AON formulation displayed ROS-triggered precision and gastrointestinal protection. The boronic ester bonds in OxbCD were hydrolyzed in a high-ROS environment (e.g., inflamed colons with elevated H₂O₂) to achieve inflammatory site-specific Ac2-26 release. The CD cavity shielded Ac2-26 from enzymatic degradation in the GI tract, enhancing oral bioavailability of Ac2-26 in the colon. Released Ac2-26 bound to FPR2 on immune cells, suppressing TNF-α/IL-1β while polarizing macrophages to a pro-resolving M2 phenotype. AON normalized dysbiosis in the colon by increasing the beneficial *Prevotellaceae* population via enhancing short-chain fatty acid production. In both acute and chronic colitis models, AON achieved improved survival rates compared with free Ac2-26, resolved inflammation and promoted epithelial regeneration (**Figure 7**) 249.

#### 4.3.2 Inorganic biomaterials

Inorganic biomaterials are defined as non-carbon-based polymers or networks comprising natural minerals (e.g., silicates, phosphates) or synthetic compounds. They display unique physicochemical properties as regenerative medicine. These materials exhibit tunable biochemical/biophysical attributes, including bioactive ion release, photothermal/magnetothermal responsiveness, and programmable degradation kinetics, and these attributes collectively enable precise control over cellular behaviors such as stem cell differentiation and apoptosis regulation. Their advantages stem from robust structural stability of inorganic materials, however, they have poor inherent biocompatibility and they are often modified with organic or bio-sourced materials to enhance cellular affinity. Therefore, seamless integration of inorganic materials into tissue-engineered scaffolds can realize targeted therapeutic delivery and microenvironment remodeling 250,251. In the Gel@fMLP/SiO₂-FasL hybrid system, SiO₂ functioned as an inorganic reinforcing agent within the organic hydrogel matrix. Through polymer chain crosslinking, SiO₂ provided critical mechanical stabilization and mitigated stress-induced deformation in the hybrid system 247.

In addition, hollow porous nanostructures of gold-based biomaterials can be synthesized via galvanic replacement reactions. These gold-based nanostructures exhibit tunable optical and physicochemical properties, and they can be versatile carriers for therapeutic agents. The gold-based nanostructures hold unique features including 1) tunable surface plasmon resonance (SPR): gold nanocages (AuNCs) can absorb near-infrared light, and deep-tissue penetration of AUNCs enables photoacoustic (PA) imaging and targeted therapy; 2) High drug-loading capacity: a porous nanostructure can efficiently encapsulate hydrophobic drugs; and 3) Biocompatibility and surface flexibility: nanostructure surfaces can be modified with targeting ligands for cell-specific delivery 252. PS-lipos-AuNC@T0901317 was prepared through multi-step engineering. Hollow porous AuNCs were synthesized via the sacrificial template method and they were coated with phospholipid monolayers via nanoprecipitation to enhance stability and realize controlled drug release. The PS ligands were inserted into the liposomal layer to mimic the surface of apoptotic cells. T0901317, an LXR agonist, was loaded into AuNC pores/lipid bilayers via solvent evaporation. This nanostructure bound to MerTK/TIM-4 receptors on macrophages by leveraging PS-mediated targeting, achieving inflammation-specific accumulation. T0901317 synergistically upregulated the MerTK expression to enhance efferocytosis of apoptotic cells and suppressed the NF-κB pathway to reduce the secretion of proinflammatory cytokines (TNF-α/IL-1β). Therefore, impaired apoptotic clearance was corrected to attenuate systemic lupus erythematosus progression in a murine model through precise immunomodulation and microenvironment remodeling 253.

#### 4.3.3 Bio-derived biomaterials

Bio-derived biomaterials are either natural materials sourced from biological systems, including polysaccharides, proteins, and polynucleotides, or genetically engineered products. These materials leverage innate biocompatibility, biodegradability, and structural versatility to mimic the native ECM, facilitating cell adhesion, proliferation, and tissue regeneration. Their advantages include low immunogenicity, enzymatic degradability into nontoxic byproducts, and tunable bioactivity through molecular design 254. They often lack the highly specific, ligand-driven signaling of purified biologicals but provide critical structural cues 255.

Protein-based biomaterials stand out for their precise molecular recognition and dynamic functionality. Natural or recombinant proteins (e.g., collagen, silk fibroin, elastin) can be engineered to possess tunable mechanical properties (e.g., elasticity, stiffness) and bioactive motifs that direct cellular behaviors 256. For example, the glycyrrhiza protein (GP) nanoparticles (GNPs) were developed to create a targeted therapeutic system by harnessing self-assembly and biocompatibility of GP. Dexamethasone (Dex) was loaded to GNPs as a “drug-carrier-in-one” to create a Dex@GNPs platform. These nanoparticles selectively bound to neutrophils via complement receptor 3 (CR3)/Fcγ receptors, hitchhiking to inflamed sites. Upon arrival, Dex@GNPs induced ROS-dependent neutrophil apoptosis to release Dex into macrophages via efferocytosis. Through this dual mechanism, macrophages were polarized toward an anti-inflammatory M2 phenotype to suppress the secretion of proinflammatory cytokines, achieving rapid resolution of acute inflammation 257.

Biomimetic nanocarriers have become an emerging drug delivery platform. They are engineered from natural cellular components, such as cell membranes or extracellular vesicles (EVs), to mimic biological functions. These cellular components include diverse EVs subtypes, including exosomes (Exo), microvesicles (MVs), and apoptotic bodies, and they inherit intrinsic proteins from source cells (e.g., adhesion molecules, signaling receptors) that enable precise targeting, immune evasion, and modulation of recipient cell behaviors. Biomimetic drug delivery systems can replicate some of the natural cellular activities through biologically functional inheritance (e.g., platelet P-selectin for monocyte binding), targeted homing through ligand-receptor interactions, and immunocloaking via membrane camouflage to extend the circulation half-life. Meanwhile, they can overcome the limitations of synthetic carriers including low bioavailability, off-target effects, and immunogenicity 258,259.

For example, EVs were harvested from HIF-1α/telomerase-expressing MSCs preconditioned with cytokines (MSC-T-HIFC). These EVs synergistically enhanced macrophage efferocytosis, shifted M1-to-M2 polarization via STAT6 activation, and inhibited collagen deposition in the Crohn's disease models 260. Its classification as a bio-derived biomaterial, distinct from a biological, is defined by its engineered nature as a carrier vehicle that orchestrates a pro-healing tissue microenvironment through the coordinated delivery of its complex cargo, not through a single, defined ligand-receptor interaction. Meanwhile, as a carrier for the bioactive lncRNA cargo, keratinocyte-derived EVs were loaded with metastasis-associated lung adenocarcinoma transcript 1 (MALAT1). These EVs competitively bound to miR-1914-3p in recipient macrophages, thereby upregulating the expression of MFGE8 and restoring the phagocytotic function via activation of the TGF-β/SMAD3 pathway, ultimately accelerating diabetic wound healing by reactivating stalled inflammation resolution 261.

Moving beyond unmodified or simply modified systems, the field has advanced toward hybridized biomimetic nanocarriers. These are engineered via strategies like membrane fusion to capitalize on the inherent properties of natural membranes, with the goal of optimizing the logistics of therapeutic delivery for enhanced targeting and immunomodulation. Platelet membrane-modified extracellular vesicles (P-EVs) were created by fusing platelet membranes with MSC-derived extracellular vesicles. They inherited platelet proteins (e.g., P-selectin), thus they selectively bound to circulating monocytes via P-selectin glycoprotein ligand-1 (PSGL-1) interaction, enabling “hitchhiking” into the ischemic myocardium with a higher retention level than unmodified EVs. Subsequently, monocytes were differentiated into M1 macrophages that endocytosed P-EVs, leading to improved phagocytosis, downregulated IL-1β/TNF-α and upregulated IL10/TGF-β, eventually enhancing M2 polarization and mitigating cardiac inflammation 262. Neutrophil apoptotic body membrane-exosomes (NAM-Exo) were generated by fusing MSC-derived exosomes with apoptotic neutrophil membranes. NAM-Exo inherited adhesion molecules (LFA-1/CD44/CD11b) from neutrophil for precise homing to the infarcted myocardium. Critically, PS on their surface mimicked the receptors on apoptotic neutrophils, thereby activating macrophage efferocytosis. This process not only enhanced macrophage uptake but also reduced inflammation via downregulating TNF-α/IL-6 and upregulating IL10/ARG1, promoted M2 polarization, inhibited cardiomyocyte apoptosis, and boosted angiogenesis supported with the formation of CD31⁺ neovessels, ultimately improving the cardiac function 263. Efferocytosis-mediated red blood cell hybrid liposomes (Effero-RLP) was a biomimetic system fabricated by coating synthetic liposomes with apoptotic red blood cell (RBC) membranes. Rosiglitazone (ROSI), a PPAR-γ agonist, was encapsulated inside liposomes. Effero-RLP leveraged the PS “eat-me” signal on apoptotic RBC membranes. Effero-RLP mimicked apoptotic cells to achieve immune-stealth delivery and evade systemic immunogenicity. Consequently, Effero-RLP induced rapid macrophage accumulation via efferocytosis and alleviated tissue inflammation 264. Meanwhile, CX3CL1-functionalized apoptotic body nanovesicles (DFO-nABs) employed a “find-eat” dual-signaling strategy to target endothelial cells (ECs) within a hypoxic wound microenvironment. Specifically, CX3CL1 bound to hypoxia-upregulated CX3CR1 receptors on ECs as a “find me” signal, while phosphatidylserine (PS) exposed on the surface interacted with PS receptors as an “eat me” signal, collectively promoting endothelial cell migration and proliferation *in vitro*. Moreover, these nanovesicles were loaded with deferoxamine (DFO), an angiogenic agent that stabilizes HIF-1α, to enhance VEGF expression and improve vascular growth. DFO-nABs has demonstrated their biocompatibility, sustained drug release, and promising efficacy in promoting angiogenesis, enhancing collagen deposition, and accelerating diabetic wound closure, thus they could be employed a therapeutic agent for chronic non-healing wounds 76.

**Figure 8:**
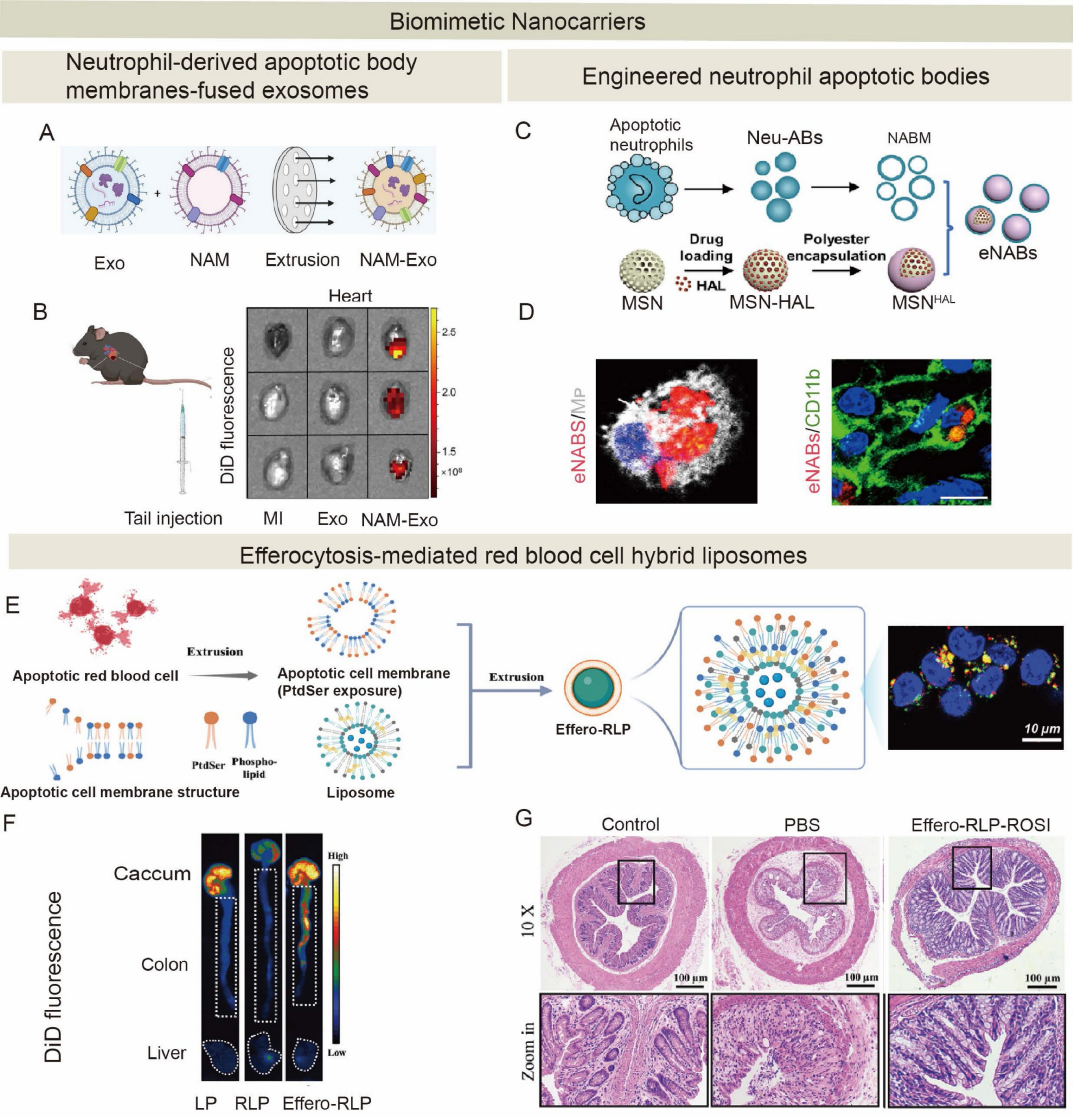
** Biomimetic nanocarriers for targeted therapeutics.** (A) Schematic illustration of the preparation of NAM-Exo. Adapted from ref.^263^. (B) *In vivo* biodistribution and infarct myocardium-targeting capability of NAM-Exo. Adapted from ref.^263^. Licensed under Creative Commons CC-BY license. (C) Fabrication process of eNABs. Adapted from ref.^265^. (D) Mechanisms for targeted therapy of myocardial infarction by eNABs. Adapted from ref.^265^ with permission of KeAi Publishing. (E) Preparation and characterization of Effero-RLP. Adapted from ref.^264^. (F) *Ex vivo* fluorescence imaging of major organs (caccum, colon, liver and blood collected from colitis mice after administration of DiD-labeled liposomes (LP), ROSI-loaded liposomes (RLP), or Effero-RLP. Adapted from ref.^264^. (G) Therapeutic efficacy of Effero-RLP-ROSI in colitis mice. Representative H&E staining images for colon tissue sections (10× overview and 40× zoomed-in views) from healthy control, PBS-treated colitis, and Effero-RLP-ROSI-treated colitis mice. Adapted from ref.^264^. Licensed under Creative Commons CC-BY license. NAM-Exo: neutrophil apoptotic membrane-fused exosomes; Exo: Exosomes; HAL: halofuginone; MSN-HAL: HAL-encapsulated mesoporous silica nanoparticles; NAMB: neutrophil apoptotic membrane blebs; Ppx: phosphatase; MI: myocardial infarction; eNABs: engineered neutrophil apoptotic bodies; Effero-RLP: efferocytosis-mediated red blood cell hybrid liposomes; PtdSer: phosphatidylserine; ROSI: rosiglitazone.

The membrane of apoptotic bodies could be harnessed as natural carriers because there are PS-mediated “eat-me” signals and inflammation-resolution cues on the membrane. The membrane of apoptotic bodies can be integrated into an inorganic-organic-biomimetic platform to achieve improved targeting efficacy through synergistic material hybridization. Engineered neutrophil apoptotic bodies (eNABs) were prepared by integrating three functionally complementary components through advanced hierarchical design to overcome sequential biological barriers in MI therapy. An inorganic mesoporous silica nanoparticle (MSN) core was used to load hexyl 5-aminolevulinate hydrochloride (HAL) due to its high loading capacity. An organic esterase-degradable polyester cap allowed enzyme-triggered intracellular HAL release within macrophages. A coating with biomimetic neutrophil apoptotic body membranes conferred inflammation-targeting via adhesion molecules LFA1/CD44/CD11b, immune evasion via the CD47 “don't eat me” signal, and macrophage-specific uptake through PS-mediated efferocytosis. This tripartite design transformed inert MSNs into a bio-intelligent therapeutic to reduce the infarct size and improve the cardiac function in a myocardial infarction model (**Figure 8**), suggesting great potential of synergistic material hybridization in targeting efferocytosis for tissue regeneration 265.

## 5. Future challenge of efferocytosis-target therapy

There are a few critical hurdles for tissue restoration via efferocytosis-targeting treatment. The primary challenge lies in the dynamic nature of efferocytosis itself. Throughout the inflammatory, reparative, and chronic phases, there are pronounced spatiotemporal variabilities in the effectiveness of efferocytosis, efferocytotic cell types, and efferocytosis-associated molecular pathways 266,267. Artificial intelligence (AI), particularly dynamic network models, are then used to synthesize these datasets to predict efferocytosis variabilities and identify critical transition points (e.g., macrophage phenotype shifts).

A second limiting factor is the complete lack of reliable real-time monitoring technologies for the investigation of efferocytosis dynamics in vivo: The current approaches for the analysis of efferocytic dynamics either rely on flow cytometric analysis or microscopy of phagocytes engulfing labeled targets, which provide only endpoint measurements with limited spatial and temporal resolution 268. Thus, the technological frontier for the next decade will be the development of smart imaging probes, e.g., enzyme-activated fluorescent nanoprobes or dual-modality tracers, which are challenged with the following design limitations: (1) sufficient specificity, e.g., targeting of apoptosis-specific signals (PS exposure or MerTK/TIM-4 receptors) with no cross-reactivity to microbial patterns; (2) high sensitivity for rare events, e.g., signal amplification strategies or super-resolution imaging in the face of limited photostability and tissue penetration; (3) biocompatibility and stability under physiological conditions without triggering immune activation; and (4) real-time monitoring of subcellular dynamics with acceptable spatial and temporal resolution (limited by slow biosensor kinetics and challenges in deep-tissue imaging).

Clinical translation of efferocytosis for tissue regeneration is also challenging due to context-dependent modulation effects of efferocytosis. An enhancement in efferocytosis can promote injury healing like myocardial infarction by clearing dead cells and mitigating necrotic burdens, while the suppression of efferocytosis is critical for interrupting efferocytosis-mediated activation of pro-fibrotic pathways in advanced liver fibrosis. Efferocytosis-targeting therapeutics should be able to exert their action in response to a specific tissue microenvironment. For example, a bifunctional antibody bridging PS on apoptotic cells and MerTK on phagocytes should be activated through a specific response to a local disease niche. The therapeutics for efferocytosis should be specific for a cell type or a cell subpopulation. The specificity can be achieved by leveraging single cell data to target disease-relevant subpopulations (e.g., Ly6C⁻ macrophages in myocardial infarction) via ligand-directed nanocarriers (e.g., anti-CD163 antibody-conjugated LNPs with phagocytosis-promoting siRNA).

Immunogenicity risks pose a significant barrier to the clinical translation of efferocytosis-targeting biologics and biomaterials, particularly for chronic conditions requiring repeated administration. Exogenous agents—including recombinant proteins (e.g., MFG-E8, Annexin V mimetics), engineered allogeneic cells (e.g., MSCs, macrophages), and synthetic nanoparticles (e.g., PLGA, gold NPs)—can trigger adverse innate and adaptive immune responses. These include neutralizing antibody production 269-271, complement activation, dendritic cell maturation, and in severe cases, cytokine release syndrome or graft rejection, ultimately reducing therapeutic efficacy and accelerating clearance 245,272-278. To mitigate these risks, a multi-pronged strategy is essential. Molecular deimmunization through computational epitope removal and full humanization of protein sequences minimizes T-cell recognition. Formulation and manufacturing improvements focus on achieving high purity, controlling critical quality attributes, and employing stabilizing excipients to prevent aggregation. For cell therapies, localized delivery or encapsulation within immunoisolative devices can circumvent systemic immune exposure. Proactive clinical strategies are equally important: MHC-II genotyping for patient stratification, co-administration of transient immunosuppressants (e.g., low-dose methotrexate) 279, and rigorous monitoring of anti-drug antibodies (ADAs) and cytokine levels allow for personalized dosing and early intervention 268. Ultimately, integrating these approaches from the molecular design stage through to clinical application is paramount for developing safe, effective, and durable efferocytosis-modulating therapies.

In face of the challenge of clinical translation, AI will be a breakthrough technology that can integrate solutions from multiple sectors. AI and machine learning (ML) provide a computational toolkit to distill meaning from complexity and speed translation by processing multi-dimensional, high-throughput data sets. Their use cases include: Firstly, ML can integrate multi-omics data (scRNA-seq, proteomics, metabolomics) to train patient-specific efferocytosis dynamics models and discover key phenotypic transitions in stratifying patient populations for targeted interventions 280. Secondly, deep learning can accelerate drug discovery by virtual screening large compound libraries for hits with modulated efferocytosis profile (e.g., MerTK, Axl, CD47 targets) or even designing new molecules de novo 281. Additionally, AI has the potential to revolutionize the design of next-generation biomaterials. By applying generative models and molecular dynamics simulations, AI can guide the discovery of smart material systems with tuned properties that are well-suited for the spatiotemporal constraints of the efferocytosis process 282. Finally, AI can aid the integration of -omic and clinical data for personalized medicines 283.

**Figure 9:**
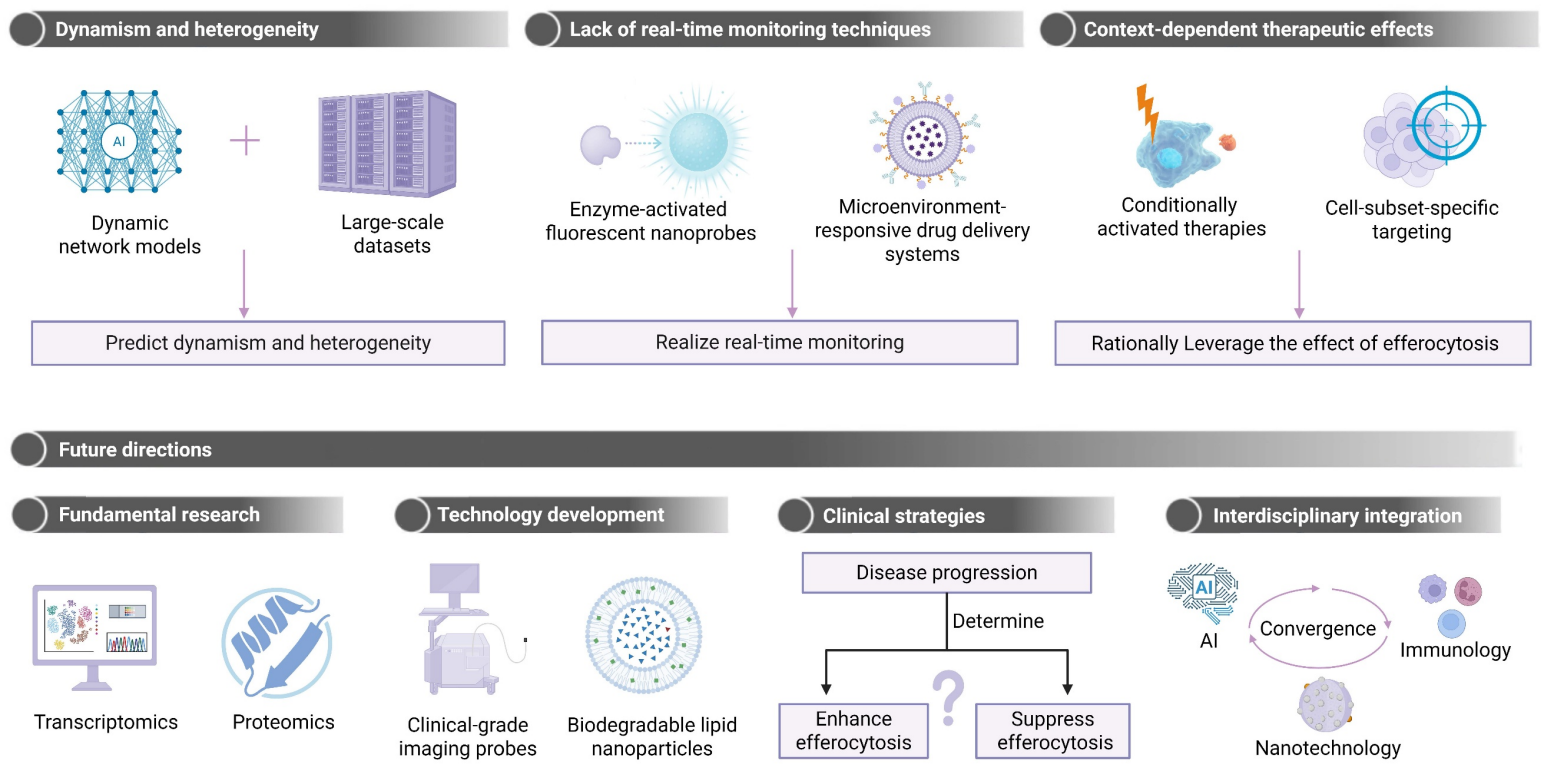
** Schematic diagram illustrating challenges in efferocytosis for tissue regeneration and AI-driven strategies for real-time monitoring and rational therapy**.

Ultimately, the road map toward multi-omics profiling of efferocytosis dynamics, enabling technologies (clinical-grade imaging probes, biodegradable LNPs), and stage-adapted clinical strategies (e.g., an enhancement of efferocytosis in the early stage vs. the suppression of efferocytosis to prevent fibrosis in the late stage) remains unclear. Therefore, the convergence of AI, nanotechnology, and immunology will expedite the attainment of dynamic sensing, precision intervention, and spatiotemporal control of tissue repair by targeting efferocytosis (**Figure 9**).

## 6. Conclusions

Efferocytosis is a central regulator of tissue regeneration, orchestrating inflammation resolution, stem cell activation, and ECM remodeling. Its dual role—resolving acute injury while potentially driving fibrosis—highlights the critical need to balance its outcomes for effective therapeutic targeting. This balance is governed by a dynamic equilibrium between regenerative and fibrotic programs, with the functional outcome determined by a critical “tipping point” involving three interconnected factors: 1) Molecular switches. The ratio of pro-efferocytic receptors (e.g., MerTK) to “don't-eat-me” signals (e.g., CD47) dictates clearance efficiency 137,139,140,147. 2) Metabolic thresholds. A phagocyte's metabolic state, shifting between oxidative phosphorylation (pro-repair) and glycolysis (pro-inflammatory), directs phenotypic polarization 57. 3) Temporal Windows. Early efferocytosis (0-72 h) is typically regenerative, while delayed clearance (> 7 days) promotes secondary necrosis and fibrosis 128.

While APIs, biologics, and smart biomaterials hold promise for enhancing efferocytosis for therapy, clinical translation is limited by concerns such as phase-specific risks to target retention, lack of target retention monitoring, and lack of real-time target retention monitoring. Solutions to these challenges likely will require integrated advances in the use of AI diagnostics with EV biomarkers, microenvironment responsive biomaterials, and multi-omics for patient stratification. Accelerating the journey from discovery to viable therapies in efferocytosis likely will require closer academia-industry partnerships to address manufacturing and regulatory pathways to fully realize the potential of efferocytosis for precision tissue regeneration.

## Figures and Tables

**Table 1:** Core mechanisms and regulatory factors of efferocytosis in tissue repair.

Stage/Key Process	Key Molecules /Axis	Function in Efferocytosis	Related Diseases	References
Recruitment phase	CX3CL1- CX3CR1	Boosting MerTK-dependent efferocytosis due to CX3CR1 deficiency	HIRI	99
		Coordinating endothelial cell migration and VEGF-mediated neovascularization	Skin wound healing	73-75
	S1P-S1PR1	Enhancing endothelial junction assembly through S1PR1-Rac1 signaling	Skin wound healing	77-79
		Promoting epithelialization by inducing keratinocyte differentiation through STAT3-mediated filaggrin expression	Skin wound healing	83,84
Recognition phase	PS	Exposing “eat-me” signal by apoptotic cells	Skeletal muscle injury	192
	CCN1	Bridging PS to αvβ3/αvβ5 integrins on phagocytes to facilitate efferocytosis by macrophages	Skin wound healing, liver injury	55,123
	MFG-E8	Bridging PS to αvβ3/αvβ5 integrins on phagocytes to modulate debris clearance	Skin wound healing, dabetic wound, MI, IBD, intestinal IRI, skeletal muscle injury	58,60,61,141,157,169-171,196
	CRT	Binding to LRP to activate ERK/PI3K pathways and promote phagocytosis	Skin wound healing	80,81
	TIM-4	Recognizing PS to promote KC efferocytosis	HIRI	100
	BAI-1	Improving efferocytosis via ELMO1/DOCK1/RAC1 signaling	Colitis	158
	CD47-SIRPα	Blocking CD47-SIRPα to restore efferocytosis and improve recovery	Liver fibrosis, MI	120,140
Digestion phase	ROS	Driving ADAM17-mediated MerTK cleavage, leading to apoptotic hepatocyte accumulation	HIRI	116
	ANXA1	Binding to FPR2/ALX on macrophages to activate AMPK signaling	Skin wound healing	66-68
	TREM2	Enhancing efferocytosis via Cox2/PGE2-mediated Rac1 activation or PI3K-AKT activation	MASH, AKI, HIRI	97,101,105,181
	MerTK	Promoting efferocytosis and tissue repair	MI, diabetic cardiomyopathy, skeletal muscle injury	93,136-139,199,200

**Table 2:** Therapeutic strategies of targeting efferocytosis for tissue regeneration.

Target Tissue	Target	Therapeutic Category	Strategy or Agent	Mechanism	Key Effects	References
Diabetic wound	PS	APIs: peptide	Ac2-26	Attenuating neutrophil infiltration, and promoting macrophage recruitment, M2 polarization and effrocytosis	Accelerating diabetic wound healing	212
	CX3CR1	Biomaterials-assisted therapy: bio-derived materials	DFO-nABs	Promoting endothelial cell migration/proliferation by targeting ECs with highly expressed CX3CR1 in a hypoxic microenvironment	Accelerating wound closure, enhancing collagen deposition, and promoting neovascularization	76
	MFG-E8	Biological products: cell therapy	MSCs	Reducing TNF-α⁺ and apoptotic cells	Accelerating diabetic wound healing	226
		Biomaterials-assisted therapy: bio-derived materials	MALAT1	Competitively binding to miR-1914-3p and upregulating MFG-E8 via TGFB1/SMAD3 activation to restore phagocytosis	Accelerating diabetic wound healing	272
	TIM-4	Biomaterials-assisted therapy: organic biomaterials	Hydrogels containing Pluronic F127, F68, and RNPs	Upregulating macrophage TIM-4 and MerTK expression to enhance efferocytosis	Accelerating diabetic wound healing	246
	SLC7A11	APIs: plant-derived	ISL, ginsenoside Rg5	Inhibiting SLC7A11 expression or impairing its function, and enhancing SLC7A11-mediated efferocytosis in DCs	Restoring the intrinsic stability of the immune microenvironment in the wound, facilitating essential remodeling of the ECM, and promoting diabetic wound healing	214,218
		Biomaterials-assisted therapy: organic biomaterials	iRGD&PS@PLGA NPs	Inhibiting SLC7A11 expression, promoting efferocytosis and inducing glycolysis-related macrophage reprogramming	Promoting diabetic wound healing	245
	FasL	Biomaterials-assisted therapy: organic biomaterials	pH-responsive hydrogels (e.g., Gel@fMLP/SiO_2_-FasL)	Recruiting neutrophils, inducing apoptosis via FasL, and promoting macrophage efferocytosis and M2 polarization	Accelerating tissue regeneration	247
	JNK-miR-21	Biomaterials-assisted therapy: organic biomaterials	MCG	Activating the macrophage efferocytosis via the JNK-miR-21 pathway	Enhancing vascularization	70
	ROS	Biomaterials-assisted therapy: bio-derived materials	Dex@GNPs	Triggering ROS-dependent neutrophil apoptosis, promoting macrophage efferocytosis and M2 polarization, and suppressing proinflammatory cytokines	Resolving acute inflammation	257
MI	PS	Biomaterials-assisted therapy: bio-derived materials	NAM-Exo	Mimicking PS exposure to enhance macrophage efferocytosis and M2 polarization and reducing inflammation	Mitigating cardiomyocyte apoptosis, enhancing angiogenesis, and improving cardiac function	263
ACLF	TREM2	APIs: plant-derived	*Scutellaria baicalensis* flavonoid baicalein	Upregulating the expression of the macrophage TREM2 receptor via TrKB-CREB1, driving M2 polarization and promoting macrophage efferocytosis	Suppressing acute inflammation and alleviating liver damage	215
IBD	PGE2	Biological products: cell therapy	hMSCs	Suppressing PGE2-dependent T-cell proliferation, and driving efferocytosis-driven macrophage reprogramming	Resolving inflammation and promoting tissue repair	227
	ROS	Biomaterials-assisted therapy: organic biomaterials	AON	Downregulating the production of proinflammatory mediators, promoting efferocytosis of apoptotic neutrophils, and enhancing phenotypic switch in macrophages	Reducing inflammation, accelerating intestinal mucosal wound healing and restoring gut homeostasis	249
Colitis	FPR2	APIs: plant-derived	COL	Enhancing efferocytosis as a biased agonist of FPR2 and promoting LAP	Resolving intestinal inflammation	216
AKI-CKD	HIF-1α	Biological products: cell therapy	TREM2^+^ macrophages	Increasing the proportion of TREM2^+^ macrophages via HIF-1α and improving efferocytosis	Mitigating tubular damage	181
Kidney damage in lupus	Mer/TIM4 receptors	Biomaterials-assisted therapy: inorganic biomaterials	PS-lipos-AuNCs loaded with T0901317	Correcting impaired apoptotic cell clearance	Attenuating systemic lupus erythematosus progression	253
Renal I/R injury	EPOR/βcR	APIs: peptide	HBSP	Promoting phagocytic clearance by TECs via upregulated EPOR/βcR	Reducing inflammation and improving renal repair	211

## Abbreviations

ACs: apoptotic cells
DAMPs: damage-associated molecular patterns
LAP: LC3-associated phagocytosis
ECM: extracellular matrix
DCs: dendritic cells
CX3CL1: chemokines CX3 C-chemokine ligand 1
CXCL12: CX C-chemokine ligand 12
S1P: lipids sphingosine 1-phosphate
LPC: lysophosphatidylcholine
ATP: adenosine triphosphate
CX3CR1: CX3 C-chemokine receptor 1
G2A: G protein-coupled receptor
P2Y2: P2Y purinoceptor 2
PS: phosphatidylserine
TIM: T-cell immunoglobulin and mucin-domain
aDGrB1: adhesion G protein-coupled receptor B1
BAI1: brain angiogenesis inhibitor-1
Gas6: growth arrest-specific gene 6
MFG-E8: milk fat globule-epidermal growth factor 8
TAM: Tyro3-Axl-MerKT
SIRPα: regulatory protein α
Rho GTPases: Ras homologous guanosine-5'-triphosphatases
ELMO: engulfment and cell motility protein
DOCK180: dedicator of cytokinesis 1
GULP1: engulfment adaptor PTB domain pontaining 1
ARP2/3: actin-related protein 2/3 complex
WAVE1: WASP-family verprolin-homologous protein 1
Rab5: Ras-related in brain protein 5
PI3KC3: class III phosphatidylinositol 3-kinase
NOX2: nicotinamide adenine dinucleotide phosphate oxidase 2
ROS: reactive oxygen species
IL-1β: interleukin -1β
TNF-α: tumor necrosis factor-α
TGF-β: transforming growth factor-β
mTORC2: mechanistic target of rapamycin complex 2
VEGF: vascular endothelial growth factor
PDGF: platelet-derived growth factor
Wnt: Wingless/INT-1
PGE2: prostaglandin E2
TSG-6: tumor necrosis factor-stimulated gene 6
IL-1α: interleukin-1α
CCN1: cellular communication network factor 1
NAD+: nicotinamide adenine dinucleotide positive
IL10: interleukin-10
STAT3: signal transducer and activator of transcription 3
NF-κB: nuclear factor kappa-light-chain-enhancer of activated B cells
PI3K/AKT: phosphoinositide 3-kinase/protein kinase B
bFGF: basic fibroblast growth factor
ICAM-1: intercellular adhesion molecule 1
SYK: spleen tyrosine kinase
ANXA1: Annexin A1
FPR2/ALX: formyl peptide receptor 2
AMPK: adenosine monophosphate-activated protein kinase
β2-AR: β2-adrenergic receptor
miR-21: microRNA-21
PTEN: phosphatase and tensin homolog deleted on chromosome 10
PDCD4: programmed cell death 4
COX-2: cycloxygenase-2
iNOS: inducible nitric oxide synthase
MCG: modified collagen gel
JNK: c-Jun N-terminal kinase
MMP-9: matrix metalloproteinase-9
CGRP: calcitonin gene-related peptide
RAMP1: receptor activity modifying protein 1
DFO-nABs: deferoxamine-functionalized apoptotic body nanovesicles
S1PR1: sphingosine-1-phosphate receptor 1
FTY720: fingolimod
CRT: calreticulin
SMAD2/3: mother against decapentaplegic homolog 2/3
SHH: Sonic Hedgehog
Gli: Glioma-associated oncogene homolog
OXPHOS: oxidative phosphorylation
AGEs: advanced glycation end products
RAGE: receptor for advanced glycation end products
sRAGE: soluble receptor for advanced glycation end products
rMFG-E8: recombinant milk fat globule-EGF factor 8
ADAM9: a disintegrin and metalloproteinase 9
PPAR-γ: peroxisome proliferator-activated receptor gamma
SLC7A11: solute carrier family 7 member 11
SAVs: Staphylococcus aureus-derived vesicles
TLR2-MyD88-p38 MAPK: toll-like receptor 2-myeloid differentiation primary response 88-p38 mitogen-activated protein kinase signaling axis
IRI: ischemia-reperfusion injury
CCR1/5: C-C chemokine receptor type 1/5
LXRα: liver X receptor α
KC: Kupffer cell
TIM-4: T-cell immunoglobulin and mucin domain-containing protein 4
RvD1: Resolvin D1
ALX/FPR2: lipoxin A4 receptor / formyl peptide receptor 2
ALD: alcohol-associated liver disease
MASH: metabolic dysfunction-associated steatohepatitis
ALOX15: arachidonate 15-lipoxygenase
HGF: hepatocyte growth factor
APAP: acetaminophen
SLPI: secretory leucocyte protease inhibitor
NAbs: natural antibodies
FcγR: Fc gamma receptor
CD11b: integrin alpha M
STING: stimulator of interferon genes
NLRP3: NOD-like receptor protein 3
AH: alcoholic hepatitis
NETs: neutrophil extracellular traps
LDNs: low-density neutrophils
NPC1: Niemann-Pick C1
FABP7: fatty acid binding protein 7
CD36: cluster of differentiation 36
MCP-1: monocyte chemoattractant protein-1
HSCs: hepatic stellate cells
FasL: Fas Ligand
ABs: apoptotic bodies
NADPH: nicotinamide adenine dinucleotide phosphate
JNK: c-Jun N-terminal kinase
ERK1/2: extracellular signal-regulated kinase 1/2
JAK: janus kinase
CDC42: cell division control protein 42
α-SMA: alpha smooth muscle actin
MI: myocardial infarction
CM: cardiomyocytes
solMER: soluble Mer tyrosine kinase
MHCIILOCCR2: major histocompatibility complex class II low expression C-C chemokine receptor type 2
CD47: cluster of differentiation 47
SIRPα: signal regulatory protein alpha
MFG-E8: milk fat globule-EGF factor 8
IL-6: interleukin-6
miR-126: microRNA-126
TREM2: triggering receptor expressed on myeloid cells 2
SMAD4: mother against decapentaplegic homolog 4
SLC25A53: solute carrier family 25 member 53
C1q: complement component 1q
TLF: T-cell leukemia/lymphoma factor
TXA2: thromboxane A2
TP: thromboxane prostanoid
YAP: yes-associated protein 1
TAZ: transcriptional co-activator with PDZ-binding motif
LGMN: legumain
LC3-II: microtubule-associated protein 1A/1B-light chain 3-II
PPAR-γ/δ: peroxisome proliferator-activated receptor gamma/delta
NGAL: neutrophil gelatinase-associated lipocalin
OPN: osteopontin
Galectin-3: galectin-3
CD206: cluster of differentiation 206
NEO1: neogenin 1
JAK1: janus kinase 1
STAT1: signal transducer and activator of transcription 1
MHC-IIhi: major histocompatibility complex class II high expression
MHC-IIlo: major histocompatibility complex class II low expression
Ly6chi: lymphocyte antigen 6 complex, locus C high
NR4A1: nuclear receptor subfamily 4 group A member 1
HAHMW: high molecular weight hyaluronan
CD44: cluster of differentiation 44
IL-17: interleukin-17
IP-10: interferon gamma-induced protein 10
GPRC5B: G protein-coupled receptor, class C, group 5, member B
EP2 receptor: E prostanoid receptor 2
cAMP: cyclic adenosine monophosphate
IBD: inflammatory bowel disease
DSS: dextran sodium sulfate
CD4+: cluster of differentiation 4 positive
TNFα: tumor necrosis factor alpha
LSECtin: liver and lymph node sinusoidal endothelial cell C-type lectin
HBEGF: heparin-binding epidermal growth factor-like growth factor
mTOR: mammalian target of rapamycin
mTORC1: mammalian target of rapamycin complex 1
TNBS: 2,4,6 trinitrobenzene sulfonic acid
rhMFG-E8: recombinant human milk fat globule-EGF factor 8
ELMO1: engulfment and cell motility 1
DOCK1: dedicator of cytokinesis 1
RAC1: Ras-related C3 botulinum toxin substrate 1
PGI2: prostaglandin I2
LXA4: lipoxin A4
15d-PGJ2: 15-Deoxy-Δ12,14-prostaglandin J2
CD300f: cluster of differentiation 300f
IECs: intestinal epithelial cells
IFN-γ: interferon gamma
ChemR23: chemerin receptor 23
mAb: monoclonal antibody
Akt: protein kinase B
EPOR: erythropoietin receptor
MRC1: mannose receptor C-type 1
YM1: chitinase-like protein 3
EPO: erythropoietin
MUC2: Mucin 2
NRBF2: nuclear receptor binding factor 2
LXR: liver X receptor
MAP: mitogen-activated protein
ABCA1: ATP-Binding Cassette Transporter Subfamily A Member 1
IL-8: interleukin-8
CCL28: C-C motif chemokine ligand 28
EMT: epithelial-mesenchymal transition
SuperMApo: super macrophage apoptotic cell-derived factors
CCL5: C-C motif chemokine ligand 5
CXCL2: C-X-C motif chemokine ligand 2
CCL22: C-C motif chemokine ligand 22
IL-1RA: interleukin-1 receptor antagonist
rmMFG-E8: recombinant mouse milk fat globule-EGF factor 8
Cth: cystathionine gamma-lyase
H_2_S: hydrogen sulfide
AKI: acute kidney injury
TECs: tubular epithelial cells
KIM-1: kidney injury molecule-1
IKK: IκB kinase
MCP-1: monocyte chemoattractant protein-1
VISTA: V-domain Ig suppressor of T cell activation
JAML: junctional adhesion molecule-like protein
IRS-1: insulin receptor substrate 1
LN: lupus nephritis
RXRα: retinoid X receptor alpha
FAO: fatty-acid-oxidation
TonEBP: Tonicity-responsive enhancer-binding protein
SCs: satellite cells
PAX7: paired box protein 7
CCR2: C-C chemokine receptor type 2
CCL2: C-C motif chemokine ligand 2
MyoD: myogenic differentiation 1
MyF5: myogenic factor 5
NFIX: nuclear factor I X
GDF3: growth differentiation factor 3
IL-10: interleukin-10
RvD1: Resolvin D1
RetSat: retinol saturase
TG2: transglutaminase 2
MyHC: myosin heavy chain
APIs: active pharmaceutical ingredients
VPA: valproic acid
HDAC: histone deacetylas
MDM2: murine double minute 2
HBSP: helix B surface peptide
EPOR/βcR: erythropoietin receptor/beta common receptor
ISL: isoliquiritigenin
TrKB: tropomyosin receptor kinase B
CREB1: cAMP response element-binding protein 1
COL: columbamine
Nr-CWS: Nocardia rubra cell wall skeleton
hMSCs: human mesenchymal stem cells
MoMF I: monocyte-derived macrophage type I
hAMTC-CM: human amniotic mesenchymal cell-conditioned medium
AAMs: alternatively activated macrophages
UUO: unilateral ureteral obstruction
HIF-1α: hypoxia-inducible factor 1 alpha
CKD: chronic kidney disease
BMDMs: bone marrow-derived macrophages
HO-1: heme oxygenase-1
Til: tiliroside
iRGD: internalizing RGD peptide
PLGA: poly(lactic-co-glycolic acid)
PKM2: pyruvate kinase isozyme M2
RNPs: ruthenium (IV) oxide nanoparticles
M-CSF: macrophage colony-stimulating factor
OxbCD: oxidation-labile β-cyclodextrin
AON: advanced oxidation-labile nanotherapy
SPR: surface plasmon resonance
PA: photoacoustic
AuNCs: gold nanocages
PS-lipos-AuNC@T0901317: phosphatidylserine-liposome-gold nanocage@T0901317
GP: glycyrrhiza protein
GNPs: glycyrrhiza protein nanoparticles
Dex: dexamethasone
HIFC: hypoxia-inducible factor cytokine
MALAT1: metastasis-associated lung adenocarcinoma transcript 1
P-EVs: platelet membrane-modified extracellular vesicles
PSGL-1: P-selectin glycoprotein ligand-1
NAM-Exo: neutrophil apoptotic body membrane-exosomes
LFA-1: lymphocyte function-associated antigen 1
Effero-RLP: efferocytosis-mediated red blood cell hybrid liposomes
RBC: red blood cell
ROSI: rosiglitazone
DFO-nABs: deferoxamine-functionalized apoptotic body nanovesicles
eNABs: engineered neutrophil apoptotic bodies
MSNs: mesoporous silica nanoparticles
HAL: hexyl 5-aminolevulinate hydrochloride
LNPs: lipid nanoparticles
siRNA: small interfering RNA
AI: artificial intelligence
